# The development of “automated visual evaluation” for cervical cancer screening: The promise and challenges in adapting deep‐learning for clinical testing

**DOI:** 10.1002/ijc.33879

**Published:** 2021-12-06

**Authors:** Kanan T. Desai, Brian Befano, Zhiyun Xue, Helen Kelly, Nicole G. Campos, Didem Egemen, Julia C. Gage, Ana‐Cecilia Rodriguez, Vikrant Sahasrabuddhe, David Levitz, Paul Pearlman, Jose Jeronimo, Sameer Antani, Mark Schiffman, Silvia de Sanjosé

**Affiliations:** ^1^ Division of Cancer Epidemiology and Genetics National Cancer Institute Rockville Maryland USA; ^2^ Information Management Services Inc. Calverton Maryland USA; ^3^ Department of Epidemiology University of Washington School of Public Health Seattle Washington USA; ^4^ US National Library of Medicine Bethesda Maryland USA; ^5^ Center for Health Decision Science Harvard T.H. Chan School of Public Health Boston Massachusetts USA; ^6^ Division of Cancer Prevention National Cancer Institute Rockville Maryland USA; ^7^ Center for Global Health, National Cancer Institute Rockville Maryland USA; ^8^ ISGlobal Barcelona Spain

**Keywords:** artificial intelligence, cervical cancer screening, clinical validation, HPV tests, visual triage

## Abstract

There is limited access to effective cervical cancer screening programs in many resource‐limited settings, resulting in continued high cervical cancer burden. Human papillomavirus (HPV) testing is increasingly recognized to be the preferable primary screening approach if affordable due to superior long‐term reassurance when negative and adaptability to self‐sampling. Visual inspection with acetic acid (VIA) is an inexpensive but subjective and inaccurate method widely used in resource‐limited settings, either for primary screening or for triage of HPV‐positive individuals. A deep learning (DL)‐based automated visual evaluation (AVE) of cervical images has been developed to help improve the accuracy and reproducibility of VIA as assistive technology. However, like any new clinical technology, rigorous evaluation and proof of clinical effectiveness are required before AVE is implemented widely. In the current article, we outline essential clinical and technical considerations involved in building a validated DL‐based AVE tool for broad use as a clinical test.

## Graphical abstract

What's new?

An emerging option for cervical cancer screening is deep learning‐based automated visual evaluation (AVE) of cervical images. Here, the authors lay out parameters for the successful development of deep learning‐based AVE. For instance, an algorithm should be trained on representative images from each of four distinct biological stages: normal cervix; infection with high‐risk HPV; precancer; and invasive cervical cancer. Characteristics that may lead to erroneous classification, such as cervicitis, should also be considered in the training. Introducing deep learning‐based methods prematurely threatens their eventual acceptance and best use.

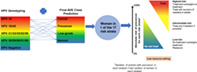

**Abbreviations**
AI: artificial intelligence
AIS: adenocarcinoma in situ
ALTS: ASCUS‐LSIL Triage Study
ASC‐US: atypical squamous cells of undetermined significance
AVE: automated visual evaluation
CIN: cervical intraepithelial neoplasia
CNN: convolutional neural network
DL: deep learning
DNA: deoxyribose nucleic acid
DR: diabetic retinopathy
DSLR: digital single lens reflex
ECC: endocervical curettage
FGS: female genital schistosomiasis
HIV: human immunodeficiency virus
HPV: human papillomavirus
HR: high‐risk
LFU: losses‐to‐follow‐up
LLETZ: large loop excision of the transformation zone
LSIL: low‐grade squamous intraepithelial lesion
ML: machine learning
NHS: Natural History Study
ROI: region of interest
SCJ: squamocolumnar junction
SIL: squamous intraepithelial lesion
TZ: transformation zone
VAT: visual assessment for treatability
VIA: visual inspection with acetic acid
WHO: World Health Organization
WLWH: women living with HIV

## 1 INTRODUCTION

Cervical cancer remains a leading cause of women's morbidity and mortality in resource‐limited settings.^1^ The World Health Organization's (WHO) global call to eliminate cervical cancer relies on high‐coverage of human papillomavirus (HPV) vaccination and screening with accurate and practical technologies to detect and treat precancers.^2^

Existing cervical cancer screening and triage technologies fall into three categories: visual, microscopic (eg, cytology) and molecular (eg, HPV testing).^3^

Visual inspection of the cervix after applying acetic acid (VIA), though widely used in low‐resource settings for primary screening or triage, is poorly reproducible across settings and not reliable in discriminating precancers from benign HPV‐related and “look‐alike” changes.^4^ Cervical cytology as performed in most low‐resource settings has had poor historical impact due to lack of infrastructure, poor quality assurance, need for repeated screening and poor follow‐up of screen positives.^5^ HPV testing is the most sensitive primary screening method for detecting precancers, thus providing long‐term reassurance for HPV‐negative women.^6^ Moreover, HPV testing is compatible with self‐collected vaginal specimens.^7^ However, to avoid overtreatment, HPV positivity is best followed by triage testing to identify the minority of HPV infections linked to precancer.^7^

Deep learning (DL)‐based automated visual evaluation (AVE) of cervical images is emerging as an alternative novel, low‐cost screening and triage solution. Machine learning (ML) is a type of artificial intelligence (AI) that uses computers to detect patterns in data without being explicitly programmed to do so.^8^ DL, inspired by the network of neurons in the human brain, is a kind of ML method that uses many layers of arithmetic operations^9^ to arrive at a model that mimics the pattern identification for which it has been trained. DL has numerous applications in medicine (eg, image recognition algorithms like AVE, automated dual‐stain cytology, diagnostic radiology and automated diabetic retinopathy [DR] screening).^10^, ^11^, ^12^ In a DL model for image recognition, information on different characteristics (eg, texture, edges and curves) associated with target of interest is gathered from individual pixels in an image through different layers. Through big data and advanced computational resources, these elements, combined in what we call an algorithm, are analyzed to provide accurate diagnosis for previously unseen images.^9^, ^13^, ^14^ AVE as an assistive technology to VIA^15^, ^16^ offers an opportunity to improve VIA to create a screening process that supports accelerated control of cervical cancer.

General reporting guidelines for clinical trials with AI‐interventions have been reported previously.^17^, ^18^ This article, however, outlines our collective view of considerations required, specifically for developing and adopting a DL‐based AVE algorithm for cervical precancer detection. We aim specifically to ensure its applicability as a well‐validated clinical test in cervical cancer screening programs globally, although most principles are likely to be applicable for any AI‐based clinical tests. The text in this article elaborates on an accompanying checklist to guide the development and validation of an effective and clinically relevant DL‐based AVE algorithm. Particularly, we wish to caution clinicians and policymakers for the need to evaluate the clinical effectiveness and applicability of those tools when they are applied in cervical cancer screening programs to avoid premature introduction (Table S1).

## 2 STEP‐WISE CONSIDERATIONS FOR AI‐BASED AVE

### 2.1 Before training the algorithm

#### 2.1.1 The indicated use of AVE

Detecting and treating precancer is the main aim of cervical screening.^19^ However, the point‐prevalence of precancer, even in previously unscreened populations, is only ~1% in the general population, and ~2.5% in the women living with HIV (WLWH).^20^ Therefore, as a general screening tool, AVE needs to detect precancers sensitively, but with the perspective that almost all screened women (>95%) will never develop cervical cancer.^21^

In contrast, among the HPV positives, the prevalence of precancer increases considerably from ~1% to >5%.^20^ Based on the well‐established role of HPV as a necessary cause in cervical carcinogenesis,^21^ together with the evidence of long‐term negative predictive value of HPV tests (virtually zero risk over 5 years),^6^ an ideal use‐case of AVE is for triage of HPV‐positive women (Box 1).

BOX 1 **AVE as a triage for HPV positives**

HPV testing for carcinogenic HPV types is the most sensitive method for cervical cancer screening, providing many years of reassurance (negative predictive value).^6^ Therefore, HPV testing is a desirable primary screening test, mainly when few screening rounds are possible.^22^, ^23^

Currently, the cost is the prohibitive factor in adopting HPV as a primary screening test in many low‐resource settings. However, based on available tests that cost <5 US dollars and take <1 hour to perform and offer partial HPV genotyping, even lower‐cost, point‐of‐care HPV tests will likely be widely available in a few years.^24^

HPV infection is too common to treat all infected women, most of whom do not need treatment, particularly given possible iatrogenic harms. Relying on negative HPV testing to reassure most women against cervical cancer risk permits public health efforts to focus on the triage of HPV‐positive women with newer technologies like HPV typing and AVE.

Risk‐informed hierarchical partial genotyping of HPV, if incorporated with minimal additional cost into HPV testing, provides important risk stratification useful for triage of HPV‐positive women.^25^ Even among the types of HPV defined as carcinogens, there are at least four distinguished categories based on the risk of invasive cancers. HPV16 (species alpha‐9) is uniquely carcinogenic with the highest risk of cervical precancer and cancer, causing ~60% of squamous cancers. HPV18 and HPV45 (species alpha‐7) cause ~15% of squamous cancers and with HPV16 also account for >90% of adenocarcinomas.^26^ The types of HPV closely related genetically to HPV16, namely, HPV31, HPV33, HPV35, HPV52 and HPV58, account for another ~15% of squamous cancers and are conceptually worth distinguishing from the lower risk, minimally carcinogenic types (HPV39, HPV51, HPV56, HPV59 and HPV68), accounting for ~5% of squamous cancers.^27^, ^28^ Of note, HPV35 is particularly pernicious for women of African origin.^29^

It is pertinent to note that if AVE is used alone for standalone primary screening, “look‐alike” confounding conditions like severe cervicitis could lead to over‐treatment^15^ of many women with benign conditions unrelated to cervical cancer. Hence AVE is used as a triage test for the relevant set of HPV‐positives. Cervical sampling for HPV testing abrades the cervix's critical transformation zone (TZ; where most cancers arise), complicating the use of AVE for triage. Fortunately, vaginal sampling, either by the woman herself or a clinician, has been convincingly shown now to be almost equivalent to clinician sampling of the cervix when a sensitive HPV DNA test is used.^30^ In addition, self‐sampling is also demonstrated to permit very high‐throughput cervical screening in a COVID‐safe manner.^20^, ^25^, ^31^

Recognizing the eventual importance of vaginal HPV testing, we aim to develop a screening strategy using HPV self‐sampling, with risk‐informed partial HPV typing^28^ and AVE. When used sequentially in combination, this will classify the woman into risk strata (of highest to lowest probability of precancer) to guide treatment and limit overtreatment.

If found to be effective, this screening strategy is envisioned by our group to be scaled up in a community‐based campaign combining: (a) screen‐and‐treat screening of mid‐adult women (ie, 25 or 30 to around 45 or 50), and (b) single‐dose vaccination of multiple birth‐cohorts of girls and younger women to induce herd protection.^32^ Such a conjoined primary and secondary prevention effort is likely to lead to accelerated cervical cancer control in low‐resource settings.

**FIGURE 1:**
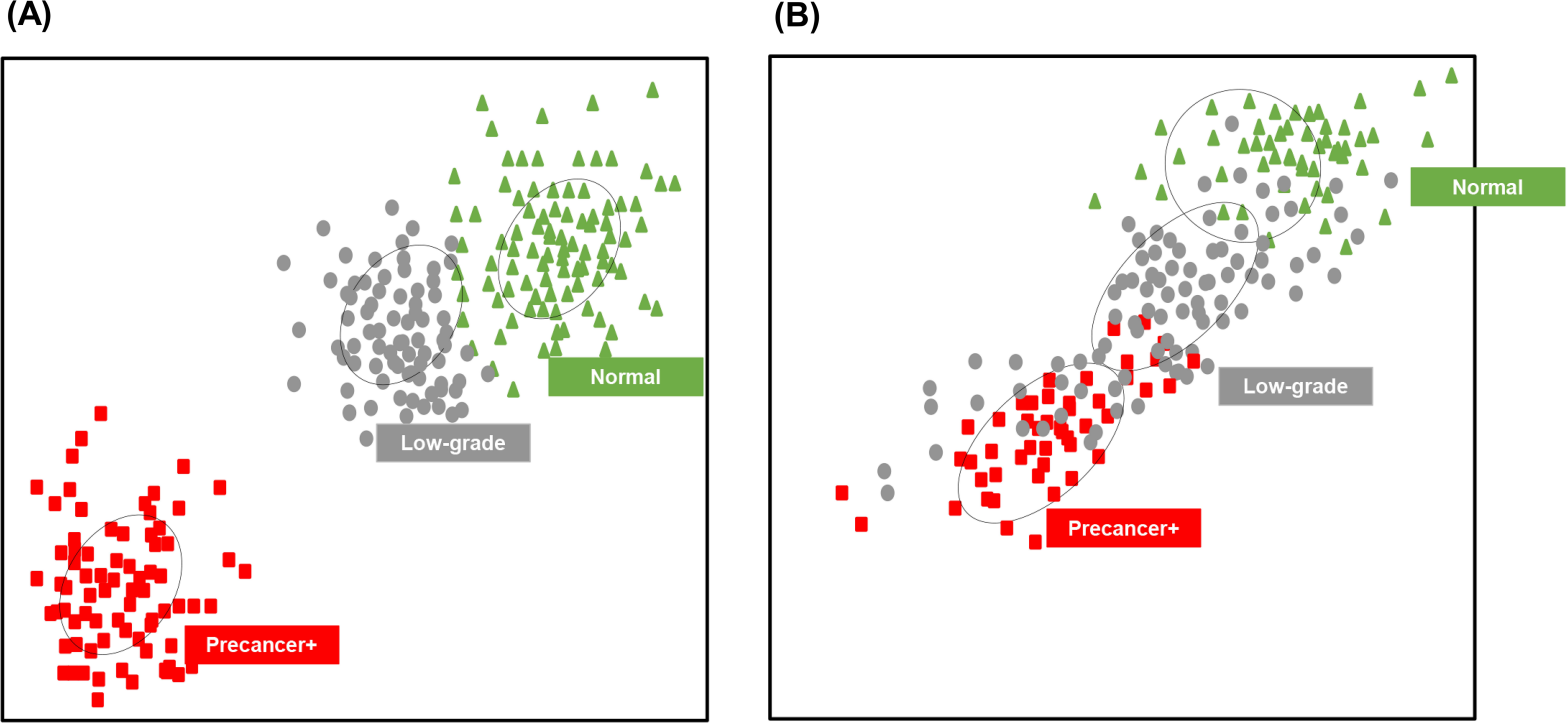
Scatterplot of algorithm scores and disease status provided in two different settings: (A) general screening and (B) triage. In (A) scores cluster in each disease state and differentiate precancer from the rest. In (B) data across disease status are sparser, and the distinction between the three disease strata is less apparent. *Source*: Graphs are conceptual and not based on real data [Color figure can be viewed at wileyonlinelibrary.com]

The prevalence of visual abnormalities and precancer further increase in a colposcopy clinic, where most women have been referred for equivocal or minor cytologic abnormalities such as HPV‐positive atypical squamous cell of undetermined significance (ASC‐US) or squamous intraepithelial lesion (SIL), respectively.^33^ Within this context, women referred for colposcopy also have an increased prevalence of cervical visual abnormalities regardless of final diagnosis. Therefore, an AVE algorithm trained for indicated use in general screening should not be assumed to be suitable for use as a tool for triage in a colposcopy setting and vice versa unless the accuracy of both approaches is explicitly demonstrated in a formal evaluation (Figures 1 and S1).

In addition, until sufficient supportive evidence accumulates regarding accuracy, reliability and portability of the method to different settings, AVE is best used as an ancillary technology to aid health workers performing VIA to improve their accuracy, rather than a standalone tool.^34^, ^35^

#### 2.1.2 Clarifying target population for using AVE

**FIGURE 2:**
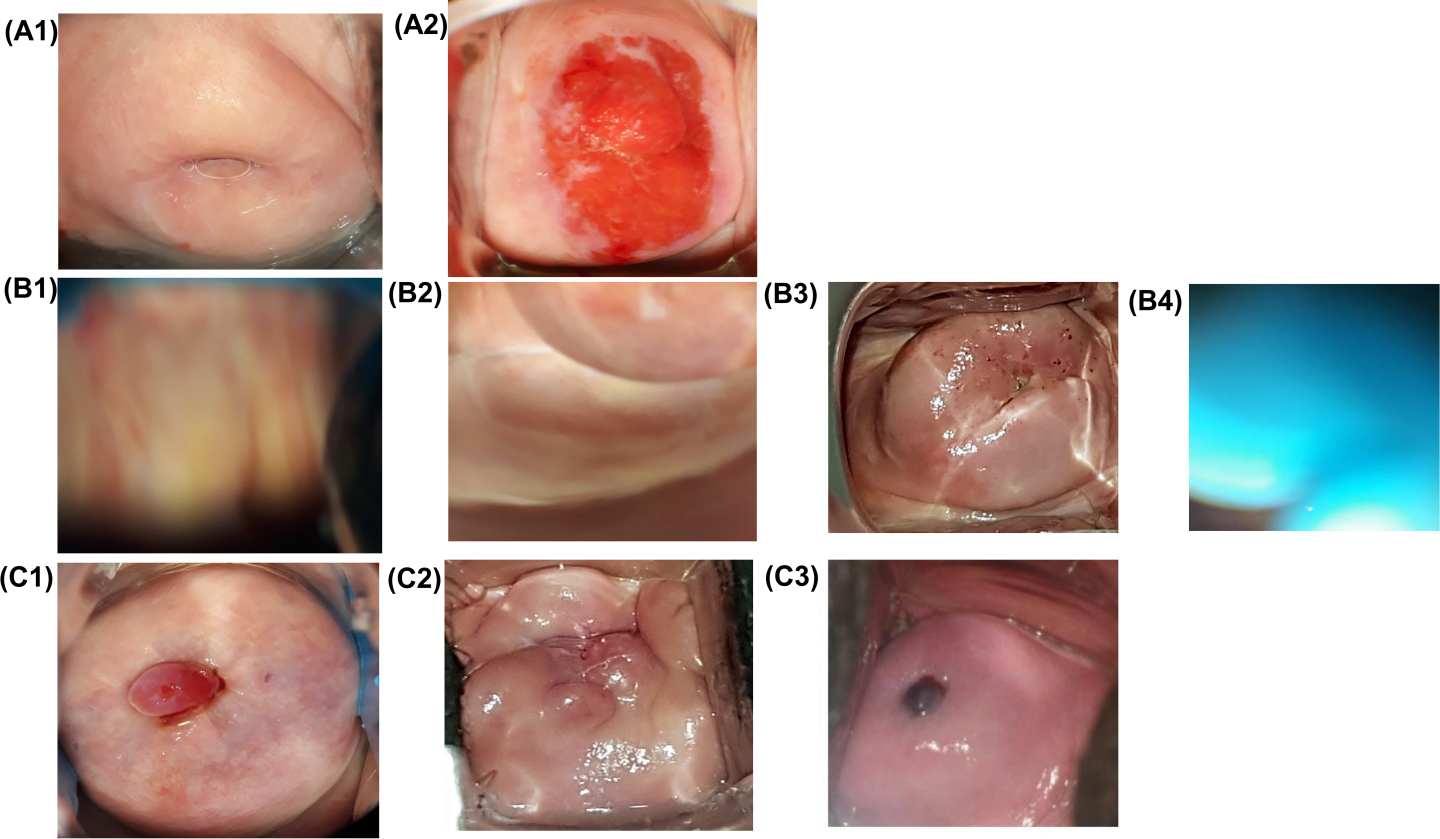
Factors affecting image interpretations by AVE. (A) The impact of age: With increasing age, the SCJ moves inside the endocervical canal, creating a pink image appearance of mature squamous epithelium and likely to provide false reassurance by negative AVE prediction. On the other hand, ectopy at younger ages is likely to lead to false‐positive results on AVE. Examples: (A1) Type III TZ (AVE prediction = negative, final diagnosis on endocervical curettage [ECC] histopathology = precancer), (A2) Ectopy (AVE prediction = precancer, Final diagnosis on ECC histopathology = normal); (B) The impact of quality: AI will give a prediction on any input, including images where the humans would even fail to identify the region of interest due to either technical factors making the image not even recognizable as a cervix. Examples: (B1) Excessive blur (AVE prediction = negative), (B2) Bad angle with the undetectable cervical os (AVE prediction = negative), (B3) speculum reflection and glare, (B4) non‐cervix image (AVE prediction = negative), OR anatomic factors obstructing the SCJ; (C) The impact of obstruction of the SCJ: Examples: (C1) Cervical polyp (AVE prediction = negative), (C2) Uterine fibroid, (C3) Menstrual blood plugging the os. *Source*: Binary classification algorithm trained on an enhanced cellphone (EVA) images globally,^39^ tested on EVA images from Project Itoju, Nigeria (unpublished results by NCI HPV‐AVE research group); Cervical image source: Desai et al^20^ [Color figure can be viewed at wileyonlinelibrary.com]

Any visual cervical screening methods, including AVE, works best when applied at an appropriate age range (eg, 25‐49 years).^36^ Within this age‐range, HPV infections are more likely to be clinically meaningful than at younger ages at which transiently detectable HPV are extremely prevalent but cancer is very rare.^37^ Moreover, prominent glandular epithelium (“ectopy” or “ectropion”), common at younger ages, may lead to false‐positive AVE predictions. Also, in mid‐adulthood compared to older ages, the squamocolumnar junction (SCJ) at which most cancers arise is frequently still fully visible,^20^ and lesions, if detected, could still be treated safely without disproportionate risk of damaging atrophic pelvic structures.^38^ Using an AVE algorithm on cervical images when the main site of cervical cancer, the SCJ, is no longer visible as occurs with aging may lead to false‐negative AVE prediction (especially when the visible epithelium covering the cervix appears completely “pink” and normal), hence, negative results in such women should be reported with caution (Figure 2A).

#### 2.1.3 Aligning the AVE classification categories with the natural history of HPV and cervical carcinogenesis

Detection of precancer is the main objective of cervical screening. However, based on the natural history, there are four biologically distinct stages in the pathogenesis of cervical cancer, which are: (a) normal cervix; (b) infection with high‐risk (HR)‐HPV (very common); (c) precancer, defined as transforming HR‐HPV infection associated with lesions with a high‐likelihood of invasion if left untreated (uncommon) and (d) invasive cervical cancer (comprising a small minority of cases compared to precancers).^37^, ^40^ Each stage in the carcinogenic process can be linked to distinct clinical management action in screening programs: (a) reassurance for women with a normal cervix; (b) triage of HR‐HPV infections; (c) treatment of precancer and (d) advanced treatment of cervical cancer.^41^ Therefore, the success of AVE can be related to its assignment of a screened individual to the proper stage linked to distinct management actions.

#### 2.1.4 Reference standard for defining the AVE classification categories

**FIGURE 3:**
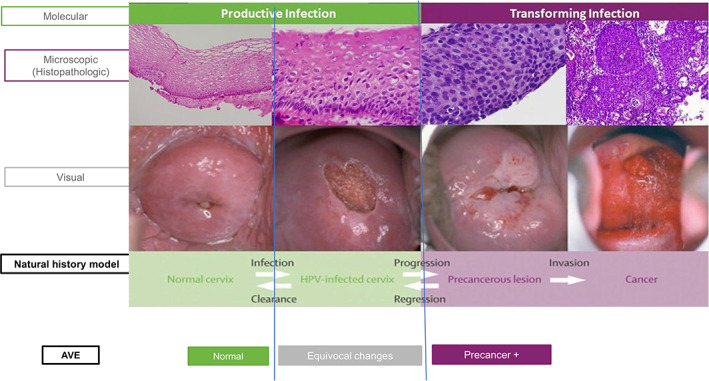
The AVE classification categories are expected to be consonant with the four biological distinct stages in the natural history and pathogenesis of cervical cancer. Reprinted with permission from Schiffman et al^21^; Histopathology image source: Desai et al^20^ [Color figure can be viewed at wileyonlinelibrary.com]

AVE needs to be trained on representative cervical images of each of the four natural history categories shown in Figure 3,^20^, ^21^ which must be defined clearly to avoid misclassification by teaching (“training”) the AVE on incorrect labels. In this regard, defining the cervical carcinogenesis stages based on nonreproducible historical grading systems (eg, dysplasia or cervical intraepithelial neoplasia [CIN] stages) is no longer optimal.^42^, ^43^, ^44^ Rather, the four stages can be defined as follows.

Invasive cervical cancer is defined histologically unless the clinical picture is so severe that surgical pathology is not obtained.

Precancer is defined stringently as a histopathologic CIN3/AIS (adenocarcinoma in situ) since most histopathologic CIN3/AIS cases contain the same HPV types found in invasive cancers.^19^, ^45^ Moreover, CIN3/AIS histopathologic diagnosis of precancer is reasonably reproducible without resorting to expensive molecular markers of cellular transformation (eg, viral methylation and viral DNA integration). Additionally, selected high‐risk histopathologic CIN2, if the diagnosis is corroborated by expert gynecologic pathologist review and accompanied with highest risk HPV‐type positivity, is likely to represent precancer.^46^ However, one needs to be cautious in including all CIN2 as a precancer target because CIN2 is a poorly reproduced diagnosis with a mixture of high‐grades and regressive low‐grades (associated with noncarcinogenic HPV types as HPV53), creating a phenocopy of early precancer. For colposcopic biopsy to be sensitive, multiple biopsies of all visible lesions (based on turning white after application of vinegar, called acetowhitening) is necessary, rather than targeting of the most severe appearing lesion. Clinician colposcopic impressions, even when performed by experienced gynecologists, are subjective and variable in distinguishing precancer from benign HPV‐related changes and “look‐alike” conditions.^47^, ^48^, ^49^ An algorithm trained on target class definitions based on human interpretation of cervical images instead of histopathologic diagnosis, particularly for “precancer” target, will be restricted by the same limitations in accuracy and intraobserver and interobserver variability as other visual methods (eg, VIA).^50^ Thus, multiple biopsies and histopathologic definition of precancer are preferable to high‐grade colposcopic impression.

However, histopathology cannot define the normal cervix, as most normal women are never thoroughly biopsied. Since the negative predictive value of the HPV test is very high, the ideal definition of “normal” (in the sense of virtually no imminent risk of cancer) will be images from confirmed HR‐HPV negative women.^6^ Alternatively, in the absence of HPV results, the absence of any acetowhitening (ie, entirely “pink” cervix) on expert review of images from women at a general screening clinic can be used to define normal because acetowhitening is a sensitive measure of the risk of precancer,^51^ and chances of finding CIN3/AIS in women at a general screening with no cervical acetowhitening is very low.^52^

Once cancer and precancer are defined histologically (and ideally virologically as well), and the normal cervix is defined visually, the remaining category can be conceived of as “HPV‐related and other equivocal changes.” Histopathology has limitations in defining this category due to subjectivity in microscopic diagnosis and biopsy placement errors (eg, targeting only the worst appearing lesions).^53^ In our experience, an algorithm not trained explicitly to recognize these “equivocal” images tends to give extremely erratic predictions on these images (Figure S2). Since it is in this “equivocal” zone where the experts also struggle the most and since the associated risk of cervical cancer is likely to be intermediate (ie, nonzero but much lower than precancer), it is desirable to train the cervical images with acetowhite changes as a separate target interposed between “normal” and “precancer” targets. Ongoing work by our group is addressing how best to include this equivocal class in training (ie, training a multiclass ordinal classifier).

### 2.2 Choosing images and metadata for training the algorithm

#### 2.2.1 Size, source and representativeness of the dataset

A typical number of learned parameters in a DL algorithm development tend to be up to millions compared to tens in a traditional multivariate model.^14^ Although it is difficult to predict the exact number, the number of representative images with truth label required to build an accurate yet generalizable AVE algorithm via DL approach, can be assumed to be hundreds or greater for each target class to achieve satisfactory disease discrimination.^54^, ^55^ It is worth recalling that, even in high‐burden settings, cervical precancer is relatively uncommon^52^; thus, ethical acquisition^35^ of accurately labeled, representative case images, is challenging.

#### 2.2.2 Image quality evaluation and pre‐exclusion

The provider's training to capture good quality images is a first step for AVE's successful application. However, when an AI‐based image recognition tool is applied in real‐world clinics, variation in the quality of images is inevitable. The image quality is affected, in addition to the user training, by the lighting (eg, external ring light vs built‐in camera flashlight, shade of white light), image capture device and postcapture processing of images by device‐specific software, anatomic variation, speculums (eg, metallic vs transparent plastic) and so on.

Without a quality check, AVE will provide a prediction for any image given to it as an input, including images not even recognizable as cervix and images with a completely obstructed region of interest (ROI) (ie, SCJ) (Figure 2B,C).^20^, ^56^ Therefore, a manual or automated gatekeeping mechanism should be in place to exclude poor‐quality images from training and evaluation to minimize false predictions.

Various parameters define the image quality, such as blur, Gaussian noise, resolution, color, angle and glare/reflections; not all affect the AVE's performance equally. The composite minimal image quality standards needed to obtain a good performance on AVE is an ongoing advanced research topic.

### 2.3 Choosing DL methods for training AVE

Training a DL algorithm is more complex than the simple explanation described previously.^15^ Multiple technical choices need to be taken while training the algorithm (Box S1),^9^, ^13^, ^14^ which may have implications for interpreting the output^54^, ^57^, ^58^ (Figure S3). The aim is: (a) to achieve accurate and reliable prediction on hold‐back images from the same database as the training set (called “internal validation”), (b) not to lose generalizability in new images from different databases (“external validation”). Ongoing work from our group is exploring the optimal DL approach to train an AVE algorithm to achieve maximum risk discrimination that has external validity.

In addition, the choice of methods has implications for time and computational speed requirements to run the algorithm. Ideally, a scalable AVE algorithm should be available to run as a standalone app (without internet) on the image‐capture device itself, providing quick (within few seconds), and real‐time predictions for on‐site patient's management to minimize loss‐to‐follow ups.

### 2.4 Validation of the output of the algorithm

#### 2.4.1 Reproducibility of AVE

The essential first parameter in assessing AVE's validity, like any medical test, is reproducibility. Like a thermometer, giving a consistent reading of body temperature on the repeated measurement of the same person, an AVE algorithm should give virtually identical outputs when asked to predict the same image repeatedly. However, in the case of near‐duplicate images (ie, images collected from a woman under the same image capture protocol consecutively), subtle changes in the numerical pixel values of the image due to changes in body or camera position may alter the AVE predictions especially for equivocal images, despite the visual similarity of the images to the human eyes. Clinically, it is confusing to the user if an AVE algorithm were to label one image as a precancer and a near‐duplicate image (or same image in a different run) as normal (Figure S4). Therefore, before its use for clinical decision‐making, AVE's robustness for near‐duplicate pairs of images should be measured and reported.^59^

#### 2.4.2 Internal validity of AVE

**FIGURE 4:**
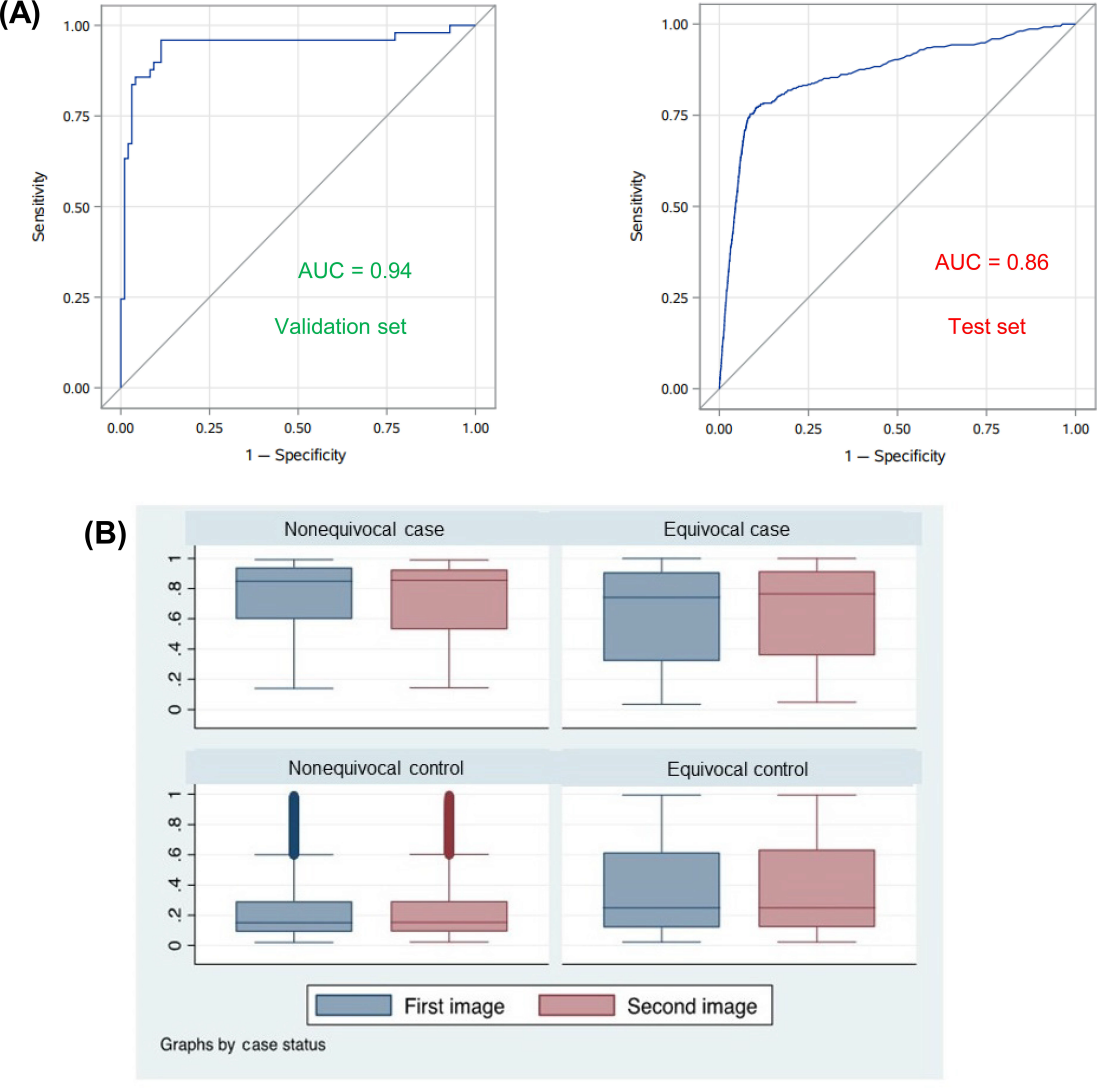
(A) AUC results for the discrimination of disease vs no disease in a validation set and a test set. Notice that the AUC value from the same study images decreases from 0.94 to 0.86 when the algorithm was tested in a hold‐back test data set images that were not used at all during the training and validation of the algorithm. *Source*: Binary classification algorithm trained on cervigram images from NHS tested on cervigram images from NHS (unpublished results by NCI HPV‐AVE research group). (B) Score values obtained in a binary classification algorithm trained on cervigram images. AVE prediction scores were presented per definite case, definite control, equivocal case and equivocal control. When using a selective set of clearly defined cases (precancers) and controls (normal), the algorithm easily discriminated between disease strata, but when adding equivocal images, as it would be in a real‐life scenario, the score distribution tended to be wider and less discriminative of diseases status. *Source*: Binary classification algorithm trained on cervigram images from NHS and ALTS tested on cervigram images from NHS and ALTS (unpublished results by NCI HPV‐AVE Research Group) [Color figure can be viewed at wileyonlinelibrary.com]

To “teach” the algorithm to recognize the target of interest, we provide it with sets of “labeled” cervical images in each target class as a “training” (to learn the features associated with the outcome of interest) and a “validation” set (to iteratively check on and optimize the algorithm's performance as part of training).^15^ It is important to note that the validation set is not a true blinded test set. A performance achieved by the algorithm on the validation set is likely to be misguiding and over‐optimistic.^58^ When the “training‐validation” set is limited, an algorithm is prone to overfitting to the image features in the “training‐validation” set and may completely fail on the third independent “hold‐back” set of previously unseen (ie, blinded) images from the same database as training and validation set.^55^, ^60^, ^61^ Therefore, it is essential to assess AVE's performance on an independent completely blinded “test” set of images not included in the “training‐validation” process (Figure 4A) to have a realistic estimate of internal validity of AVE on a dataset.

In addition, it is important to include a realistic set of images in the “test” set on which the performance is finally evaluated. For example, we may observe good case‐control discrimination by AVE on a “restricted” test set, including only the clearest examples of high‐grade cases (CIN3) and HPV‐negative controls. However, it is important to realize that many cervix images will fall into an equivocal intermediate zone, including HPV infection, cervicitis and low‐grade changes. Without examining the discrimination and reproducibility achieved by AVE in this intermediate zone of “not so clear” case or control assigment (ie, due to noisy data) where even the expert colposcopists struggle the most, the promising claims about the algorithm's capacity could be misleading for real‐life implementation (Figure 4B).

A critical aspect to evaluate the algorithm's performance is choosing the appropriate statistical approach. First, AVE class predictions can be compared against the reference standard (eg, histopathology) in a comprehensive independent test set. Second, the AVE, to be worth adopting, would ideally demonstrate consistently superior performance to the existing standard of care (eg, unaided VIA as practiced in the setting), and at least noninferior performance to the expert clinicians (eg, colposcopists). Of note, AVE is not limited in performance by human factors such as fatigue, mental stress and so on; hence is theorized to have lower intraobserver variability in addition to lower interobserver variability than VIA and colposcopy, leading to higher consistency.

#### 2.4.3 External validity (generalizability) of AVE and avoiding overfitting

Verifying an AVE algorithm's performance is a two‐step process. Testing the algorithm's performance (achieving accurate predictions without overfitting) on an independent “test” set of images derived from the same source as the training set (called “internal validation”) is a crucial first step,^15^ but not a final benchmark. This testing set will be limited by the same “finite” representation and idiosyncratic random variations as in the “training” set. Thus, the process does not reflect true validation of an algorithm in terms of how it will perform in actual clinical practice with “infinite” variations in patient characteristics, user training and image capture protocols.^9^ For example, an AVE algorithm that is overfitted to a particular set of images from a clinic^15^ will learn to recognize random (ie, nonrelevant) variations in the particular training set that distinguish precancer from normal, but these distinctions are not necessarily generalizable to other settings (eg, images from different clinics captured under different light sources by different providers) to distinguish patterns associated with precancer detection^55^, ^60^, ^61^ (Figure S5). Therefore, to assess true generalizability, one needs to evaluate the AVE algorithm's performance on a diverse set of images from various clinical settings worldwide. In addition, ideally, multiple independent formal efficacy assessments should demonstrate replicability of the results.^34^, ^62^

#### 2.4.4 Device portability of AVE

The AVE algorithm works on a pixel‐level (ie, trying to compare and contrast the differences in the pixels on the images and what impact this can have in classifying them). Therefore, an AVE algorithm trained on images from one type of image capture device tends to be overfitted to the features (ie, pixel patterns) of the particular device and works well only on that device.^55^, ^63^ A device‐agnostic AVE algorithm (eg, the current advanced state of facial recognition, likely achieved due to millions of images available for training) that can read accurately across different image capture devices (Figure S6) with minimal adaptation is a critical subcomponent of AVE's generalizability. Such a device‐agnostic algorithm does not yet exist for evaluation of cervical images and is a subject of active research. Unless efforts to develop a device‐agnostic algorithm are successful, a dedicated image capture device or devices, with algorithms trained with their image types, will need to be used to ensure accurate and time‐stable AVE performance.

#### 2.4.5 Anatomical and biologic confounding factors and effect modifiers for AVE

Several patient characteristics may contribute to the erroneous classification of a given image by AVE (Figure S7).^15^ For example, ectopy among young women may result in a high AVE severity class prediction due to the ruddy glandular epithelium extending onto the ectocervix. Similarly, severe cervicitis, female genital schistosomiasis (FGS) can be misclassified as precancer, and certain noncarcinogenic HPV types (eg, HPV71) with no relationship to cervical cancer may cause warty cervical lesions resulting in erroneous high AVE severity class prediction.

The co‐existence of human immunodeficiency virus (HIV) infection with HPV infection is probably the most important known difficulty in AVE classification. WLWH, due to shared risk factors for acquisition of infection, have 2‐fold increased likelihood of acquiring HPV. Due to HIV‐associated immunosuppression, they have a greatly elevated risk of persistent HPV and precancer, leading to a 6‐fold increased risk of cervical cancer compared to HIV‐negative women.^64^, ^65^ Precancerous lesions tend to be more severe in WLWH,^66^ affecting the anogenital epithelium more widely. WLWH also have a high risk of co‐infection with other cervical sexually transmitted infections, which can cause cervicitis that may impact visual appearance of the cervix.

HIV, FGS and cervicitis are highly prevalent in areas with a high burden of cervical cancer as well (eg, schistosomiasis and HIV in sub‐Saharan Africa, cervicitis in India). Therefore, it is critical to evaluate the need to train the AVE to control for these factors as much as possible and whether to have a subgroup‐specific AVE is needed. Beyond the scope of this discussion, the use of DL algorithms to diagnose FGS is under consideration.

#### 2.4.6 Risk prediction: “calibration” of AVE

**FIGURE 5:**
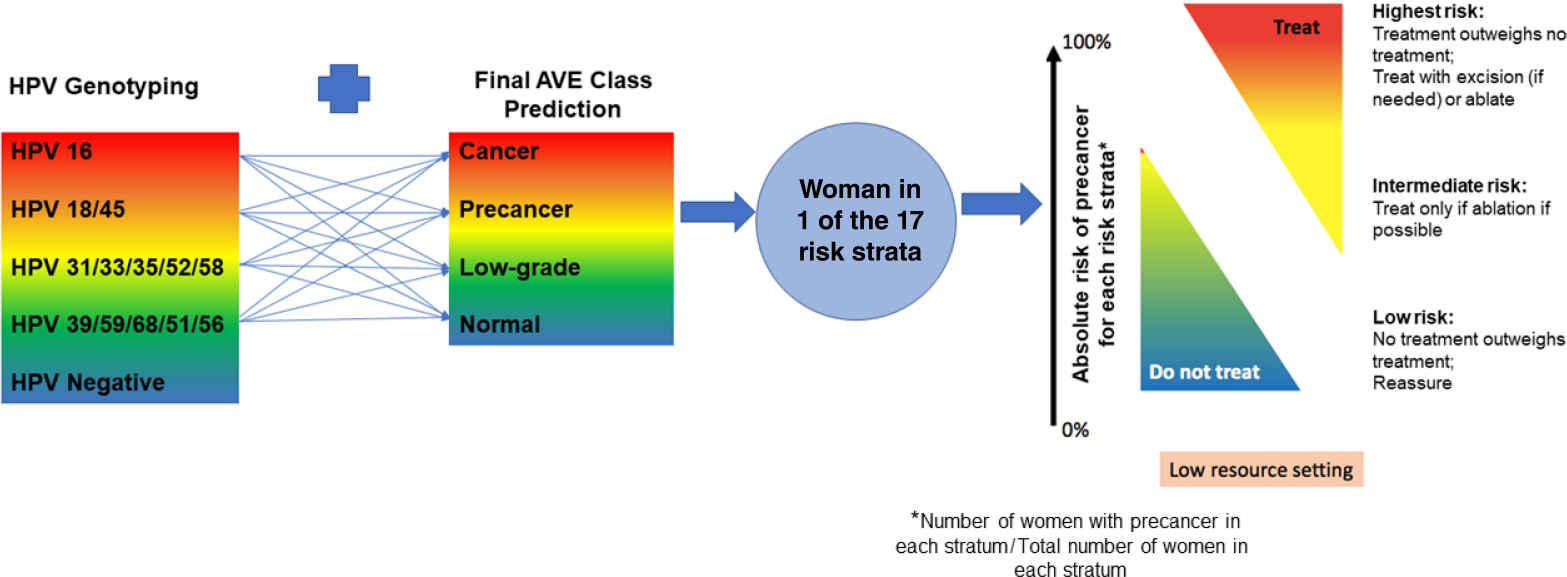
A recommended approach for cervical cancer screening based on HPV genotyping and AVE. HPV extended genotype provides a risk stratification that, when added to the AVE class label prediction, provides 17 risk strata. When each stratum is calibrated to represent the absolute probability of a woman having a precancer (ie, risk), a direct risk‐based clinical management decision can be taken tailored to resources availability. Reprinted with permission from Wentzensen et al^3^ [Color figure can be viewed at wileyonlinelibrary.com]

Before adopting any DL‐based diagnostic tool in clinical practice, the clinician should ask what is the tool measuring (ie, output) and the limitations of its interpretation for clinical decision‐making. The major goal of AVE, ideally, is to directly predict the risk (conceptually a continuous probability from 0 to 1) of a woman having a precancer today while having some reassurance (ie, negative prediction) for the future.^67^ However, the current classifier AVE algorithm approach is trained to predict discrete target classes (eg, histopathologic cancer or precancer, low‐grade, normal). Such a classifier AVE provides a score associated with each target class it is trained to predict. However, it is important to understand that these scores themselves are not true risk estimates (ie, woman with a “raw” score of 0.9 associated with the “precancer” class does not necessarily have a 90% probability of precancer) and are not reliably portable.^8^, ^68^ In order to obtain a clinically meaningful, reliable and portable estimate of the true risk of precancer from a classification network, the final AVE class label prediction needs to be translated into a risk value (ie, observed total number of women with precancer out of the total number of women with a given class prediction should match the expected number of women with precancer based on the absolute risk prediction for the given class, for example, 90 observed women with precancer out of 100 for the precancer risk of 90% for a precancer class prediction), taking into account other co‐factors (ie, age, HIV status, HPV status, HPV types, etc), if available (Figure 5, ^3^), to accurately risk‐discriminate low‐risk and high‐risk individuals for risk‐based clinical management. Such multivariate models are not yet validated.

#### 2.4.7 Predicting immediate vs future risk

AVE algorithms have been commonly trained cross‐sectionally on the woman's present status and images, rather than a longitudinal set of images per woman, and are therefore likely to only predict a prevalent risk of precancer. At present, it is not known for how long a negative AVE test confers reassurance. It is unlikely to be for as long as an HPV test.^6^

### 2.5 Field implementation

The considerations described here are mainly focused on the technical efficacy of AVE. When scaled‐up for implementation, outside the research settings, even well‐validated algorithms will have many challenges (eg, data privacy, patient acceptability and provider training) as observed in other medical fields.^63^, ^69^, ^70^ For example, even a highly accurate DR screening algorithm inside a computer lab has been documented to have failed in the field clinics due to practical challenges.^71^ Some of these challenges present valuable parallels to the AVE implementation work. For example, the DR algorithm could not read a high proportion of images due to poor quality attributed to variation in lighting conditions across the field clinics, differentially affecting the retinal dilations.^70^, ^71^ Also, it was sometimes impossible to take a single image capturing the entire field of view (eg, retina), leading to a failed prediction by the algorithm.^70^ These image quality issues lead to a similar dilemma as in AVE of balancing the risk of predictions based on imperfect data against the risk of inaction from losses‐to‐follow‐up (LFU) during referrals.^70^ There is a balance between efforts to improve image quality by users against making the algorithm robust enough to tolerate “less than perfect” images. The delayed or failed image analysis on a cloud‐based DR algorithm due to poor internet connectivity at the field clinics is another parallel with AVE, confirming our group's insistence on the absolute need for the AVE algorithm to work off of a local hardware with sufficient processing power without internet connectivity.^70^, ^71^ The challenges encountered in developing a robust and reliable DR algorithm also have many analogs for the AVE development effort. Some of these challenges are: generating reproducible ground truth labels with high interobserver agreement among the experts for training the algorithm, particularly for the classes with high interclass similarities (eg, hard vs soft exudates)^72^; difficulties in detecting lesions in the presence of noise (eg, optical reflections) and commonly encountered nonlesion structures (nerve fiber reflections, vessel reflections and drusen)^72^; and developing a generalizable algorithm that could work accurately across inevitable common variations in the clinical environment (eg, images collected from multiple centers on machines ranging from smartphone cameras to high‐end fundoscopes).^73^

The main important considerations specific for implementation of AVE for cervical cancer screening are human resource capacity‐building to manage screen‐positive women detected by AVE, developing data management systems to support tracking women needing referral and cost‐effectiveness analysis to evaluate AVE's impact in real‐life programs.

It is important to emphasize that to prevent cancer we need to detect precancer lesions and treat them adequately. Absence of treatment is a major and unfortunately very common reason for screening program's failure. For women requiring treatment, thermal‐ablation using a battery‐operated mobile device is currently the most portable option given that it is safe, effective, affordable and does not require sophisticated equipment.^74^ However, because not all women are eligible for thermal‐ablation due to abnormalities or benign changes on the cervix,^75^ local providers will need to identify which women require referral for further evaluation for more invasive treatments (eg, conization, Large Loop Electrosurgical Excision of the Transformation Zone [LLETZ]), unavailable in many resource‐limited settings. For providers, this assessment is prone to variability and challenges.^76^ DL‐based AVE based on expert reviews of cervical images is under development to predict a woman's eligibility for treatment with ablation; an initial pilot suggesting good performance.^16^

## 3 CONCLUSIONS

DL‐based AVE of the cervical image is a promising but still evolving clinical test. Even though the inner workings of DL remain obscure, DL‐based AVE, in the end, is no different from any other clinical diagnostic test. Since the limitations of the DL described here might not be fully appreciated by end‐users, the onus lies on the developer of an AI‐based device to make the subtle issues explicit, particularly in the less regulated markets. Raising awareness and knowledge of the goodness‐of‐fit and limitations of DL‐based AVE among end users is critical to improve clinical practice. Nonetheless, some AVE‐type products are already being marketed without substantial documentation of effectiveness.^77^, ^78^ Thus, in line with the WHO guidance,^35^ we maintain that premature introduction of AI‐based methods, without transparency and accountability, threatens their eventual acceptance and best use.

## CONFLICT OF INTEREST

DL is the co‐founder of MobileODT. In the last 3 years, he was an executive into company and sat on the board of directors. He is no longer with the company, but still own some stock. He is currently the owner of Imaging and Analytics Consulting, Ltd., a small consulting company based in Israel. His wife is the owner of DL Analytics, LLC, a small business based in California. Other authors have nothing to declare.

## AUTHOR CONTRIBUTIONS

Kanan T. Desai, Silvia de Sanjosé and Mark Schiffman contributed substantially to the conception and design of the study. Ana‐Cecilia Rodriguez, Brian Befano, David Levitz, Didem Egemen, Helen Kelly, Jose Jeronimo, Julia Gage, Nicole Campos and Paul Pearlman contributed to the interpretation and critical thinking. Sameer Antani and Zhiyun Xue contributed to the DL algorithm development. Kanan T. Desai drafted the manuscript. All authors provided critical revision of the article and provided final approval of the version to publish.

## Supporting information


**Appendix S1** Supporting Information.

## References

[1] Arbyn M , Weiderpass E , Bruni L , et al. Estimates of incidence and mortality of cervical cancer in 2018: a worldwide analysis. Lancet Glob Heal. 2020;8(2):e191‐e203. 10.1016/S2214-109X(19)30482-6 PMC7025157 31812369

[2] Cervical Cancer: An NCD We Can Overcome [Internet]. https://www.who.int/dg/speeches/detail/cervical-cancer-an-ncd-we-can-overcome. Accessed July 18, 2020.

[3] Wentzensen N , Schiffman M , Palmer T , Arbyn M . Triage of HPV positive women in cervical cancer screening. J Clin Virol. 2016;76:S49‐S55. 26643050 10.1016/j.jcv.2015.11.015 PMC4789103

[4] Catarino R , Schäfer S , Vassilakos P , Petignat P , Arbyn M . Accuracy of combinations of visual inspection using acetic acid or lugol iodine to detect cervical precancer: a meta‐analysis. BJOG. 2018;125(5):545‐553. 28603909 10.1111/1471-0528.14783

[5] Stoler MH , Schiffman M . Interobserver reproducibility of cervical cytologic and histologic interpretations: realistic estimates from the ASCUS‐LSIL triage study. J Am Med Assoc. 2001;285(11):1500‐1505. 10.1001/jama.285.11.1500 11255427

[6] Gage JC , Schiffman M , Katki HA , et al. Reassurance against future risk of Precancer and cancer conferred by a negative human papillomavirus test. J Natl Cancer Inst. 2014;106(8):dju153. 25038467 10.1093/jnci/dju153 PMC4111283

[7] Arbyn M , Smith SB , Temin S , Sultana F , Castle P . Detecting cervical precancer and reaching underscreened women by using HPV testing on self samples: updated meta‐analyses on behalf of the collaboration on self‐sampling and HPV testing. BMJ. 2018;363:4823. 10.1136/bmj.k4823 PMC6278587 30518635

[8] Long LR . Introduction to Neural Networks and Deep Learning [Unpublished material]. Bethesda, MD: US National Library of Medicine; 2021.

[9] Liu Y , Chen PHC , Krause J , Peng L . How to read articles that use machine learning: users' guides to the medical literature. JAMA. 2019;322:1806‐1816. 31714992 10.1001/jama.2019.16489

[10] Yu KH , Beam AL , Kohane IS . Artificial intelligence in healthcare. Nat Biomed Eng. 2018;2:719‐731. doi:10.1038/s41551-018-0305-z 31015651

[11] Yu K , Hyun N , Fetterman B , et al. Automated cervical screening and triage, based on HPV testing and computer‐interpreted cytology. J Natl Cancer Inst. 2018;110:1222‐1228. 29659930 10.1093/jnci/djy044 PMC6454428

[12] Wentzensen N , Lahrmann B , Clarke MA , et al. Accuracy and efficiency of deep‐learning‐based automation of dual stain cytology in cervical cancer screening. J Natl Cancer Inst. 2021;113(1):72‐79. 32584382 10.1093/jnci/djaa066 PMC7781458

[13] Choi RY , Coyner AS , Kalpathy‐Cramer J , Chiang MF , Campbell JP . Introduction to machine learning, neural networks, and deep learning. Transl Vis Sci Technol. 2020;9(2):14. 10.1167/tvst.9.2.14 PMC7347027 32704420

[14] Chartrand G , Cheng PM , Vorontsov E , et al. Deep learning: a primer for radiologists. Radiographics. 2017;37:2113‐2131. doi:10.1148/rg.2017170077 29131760

[15] Hu L , Bell D , Antani S , et al. An observational study of deep learning and automated evaluation of cervical images for cancer screening. J Natl Cancer Inst. 2019;111:923‐932. 30629194 10.1093/jnci/djy225 PMC6748814

[16] Guo P , Xue Z , Jeronimo J , et al. Network visualization and pyramidal feature comparison for ablative treatability classification using digitized cervix images. J Clin Med. 2021;10(5):953. 33804469 10.3390/jcm10050953 PMC7957626

[17] Liu X , Cruz Rivera S , Moher D , et al. Reporting guidelines for clinical trial reports for interventions involving artificial intelligence: the CONSORT‐AI extension. Lancet Digital Health. 2020;2:e537‐e548. 33328048 10.1016/S2589-7500(20)30218-1 PMC8183333

[18] Cruz Rivera S , Liu X , Chan AW , et al. Guidelines for clinical trial protocols for interventions involving artificial intelligence: the SPIRIT‐AI extension. Lancet Digit Health. 2020;2:e549‐e560. 33328049 10.1016/S2589-7500(20)30219-3 PMC8212701

[19] Castle PE , Pierz AJ , Adcock R , et al. A pooled analysis to compare the clinical characteristics of human papillomavirus‐positive and ‐negative cervical precancers. Cancer Prev Res. 2020;13(10):829‐840. 10.1158/1940-6207.CAPR-20-0182 32655005

[20] Desai KT , Ajenifuja KO , Banjo A , et al. Design and feasibility of a novel program of cervical screening in Nigeria: self‐sampled HPV testing paired with visual triage. Infect Agent Cancer. 2020;15:60. 33072178 10.1186/s13027-020-00324-5 PMC7556552

[21] Schiffman M , Castle PE , Jeronimo J , Rodriguez AC , Wacholder S . Human papillomavirus and cervical cancer. Lancet. 2007;370:890‐907. 17826171 10.1016/S0140-6736(07)61416-0

[22] Gage JC , Katki HA , Schiffman M , et al. The low risk of precancer after a screening result of human papillomavirus‐negative/atypical squamous cells of undetermined significance papanicolaou and implications for clinical management. Cancer Cytopathol. 2014;122:842‐850. 25045058 10.1002/cncy.21463

[23] World Health Organization (WHO) . WHO Guideline for Screening and Treatment of Cervical Pre‐Cancer Lesions for Cervical Cancer Prevention. Geneva, Switzerland: WHO; 2021. 34314129

[24] Zhang W , Du H , Huang X , et al. Evaluation of an isothermal amplification HPV detection assay for primary cervical cancer screening. Infect Agents Cancer. 2020;15:65. 10.1186/s13027-020-00328-1 PMC7583687 33110442

[25] Ajenifuja KO , Belinson J , Goldstein A , Desai K , de Sanjosé S , Schiffman M . Designing low‐cost, accurate cervical screening strategies that take into account COVID‐19: a role for self‐sampled HPV typing. Infect agents Cancer. 2020;15:61. 10.1186/s13027-020-00325-4 PMC7556607 33072179

[26] Tjalma WAA , Stoler MH , Depuydt CE , Wright TL . Don't forget HPV‐45 in cervical cancer screening. Am J Clin Pathol. 2012;137(1):161‐163. 22180491 10.1309/AJCPYB6C4HIMLZIX

[27] de Sanjosé S , Serrano B , Tous S , et al. Burden of human papillomavirus (HPV)‐related cancers attributable to HPVs 6/11/16/18/31/33/45/52 and 58. JNCI Cancer Spectr. 2018;2(4):pky045. 31360870 10.1093/jncics/pky045 PMC6649711

[28] Demarco M , Hyun N , Carter‐Pokras O , et al. A study of type‐specific HPV natural history and implications for contemporary cervical cancer screening programs. EClinicalMedicine. 2020;22:100293. 32510043 10.1016/j.eclinm.2020.100293 PMC7264956

[29] Pinheiro M , Gage JC , Clifford GM , et al. Association of HPV35 with cervical carcinogenesis among women of African ancestry: evidence of viral‐host interaction with implications for disease intervention. Int J Cancer. 2020;147:2677‐2686. 32363580 10.1002/ijc.33033 PMC11090644

[30] Arbyn M , Smith SB , Temin S , Sultana F , Castle P . Detecting cervical precancer and reaching underscreened women by using HPV testing on self samples: updated meta‐analyses. BMJ. 2018;5:363. 10.1136/bmj.k4823 PMC6278587 30518635

[31] Goldstein A , Lei Y , Goldstein L , et al. A rapid, high‐volume cervical screening project using self‐sampling and isothermal PCR HPV testing. Infect Agent Cancer. 2020;15:64. 33106753 10.1186/s13027-020-00329-0 PMC7579849

[32] Kreimer AR , Sampson JN , Porras C , et al. Evaluation of durability of a single dose of the bivalent HPV vaccine: the CVT trial. J Natl Cancer Inst. 2020;112(10):1038‐1046. 32091594 10.1093/jnci/djaa011 PMC7566548

[33] Katki HA , Schiffman M , Castle PE , et al. Five‐year risks of CIN 3+ and cervical cancer among women with HPV testing of ASC‐US pap results. J Low Genit Tract Dis. 2013;17:S36‐S42. 23519303 10.1097/LGT.0b013e3182854253 PMC3616508

[34] Topol EJ . High‐performance medicine: the convergence of human and artificial intelligence. Nat Med. 2019;25:44‐56. doi:10.1038/s41591-018-0300-7 30617339

[35] World Health Organization (WHO) . Ethics and governance of artificial intelligence for health. Geneva, Switzerland: WHO; 2021.

[36] Ren H , Jia M , Zhao S , Li H , Fan S . Factors correlated with the accuracy of colposcopy‐directed biopsy: a systematic review and meta‐analysis. J Investig Surg. 2020;34:1‐9. 10.1080/08941939.2020.1850944 33377808

[37] Schiffman M , Doorbar J , Wentzensen N , et al. Carcinogenic human papillomavirus infection. Nat Rev Dis Prim. 2016;2(1):16086. 27905473 10.1038/nrdp.2016.86

[38] Cho A , Park S , Park S , et al. Hemoperitoneum: a complication of loop electrosurgical excision procedure. Obstet Gynecol Sci. 2019;62(2):138‐141. 30918883 10.5468/ogs.2019.62.2.138 PMC6422840

[39] Xue Z , Novetsky AP , Einstein MH , et al. A demonstration of automated visual evaluation of cervical images taken with a smartphone camera. Int J Cancer. 2020;147(9):2416‐2423. doi: 10.1002/ijc.33029 PMC11977665 32356305

[40] Campos NG , Demarco M , Bruni L , et al. A proposed new generation of evidence‐based state‐transition models to inform global control of cervical cancer. Prev Med. 2021;144:106438. 33678235 10.1016/j.ypmed.2021.106438 PMC8041229

[41] Schiffman M , Wentzensen N . A suggested approach to simplify and improve cervical screening in the United States. J Low Genit Tract Dis. 2016;20(1):1‐7. 26704326 10.1097/LGT.0000000000000170 PMC4692178

[42] Schiffman M . The need for forward‐looking decision analyses to guide cervical cancer prevention. Cancer Epidemiol Biomarkers Prev. 2011;20(2):219‐220. 21300615 10.1158/1055-9965.EPI-10-1130

[43] Guan P , Howell‐Jones R , Li N , et al. Human papillomavirus types in 115,789 HPV‐positive women: a meta‐analysis from cervical infection to cancer. Int J Cancer. 2012;131(10):2349‐2359. 22323075 10.1002/ijc.27485

[44] Schiffman M , de Sanjose S . False positive cervical HPV screening test results. Papillomavirus Res. 2019;7:184‐187. 31029852 10.1016/j.pvr.2019.04.012 PMC6514435

[45] Castle PE , Schiffman M , Wheeler CM , Wentzensen N , Gravitt PE . Human papillomavirus genotypes in cervical intraepithelial neoplasia grade 3. Cancer Epidemiol Biomarkers Prev. 2010;19(7):1675‐1681. 20615885 10.1158/1055-9965.EPI-10-0251 PMC2901898

[46] Carreon JD , Sherman ME , Guillén D , et al. CIN2 is a much less reproducible and less valid diagnosis than CIN3: results from a histological review of population‐based cervical samples. Int J Gynecol Pathol. 2007;26(4):441‐446. 17885496 10.1097/pgp.0b013e31805152ab

[47] Jeronimo J , Schiffman M . Colposcopy at a crossroads. Am J Obstet Gynecol. 2006;195(2):349‐353. 16677597 10.1016/j.ajog.2006.01.091

[48] McCluggage WG , Bharucha H , Caughley LM , et al. Interobserver variation in the reporting of cervical colposcopic biopsy specimens: comparison of grading systems. J Clin Pathol. 1996;49(10):833‐835. 8943751 10.1136/jcp.49.10.833 PMC500779

[49] Rodríguez AC , Morera LA , Bratti C , et al. Performance of direct visual inspection of the cervix with acetic acid and magnification in a previously screened population. J Low Genit Tract Dis. 2004;8(2):132‐138. 15874851 10.1097/00128360-200404000-00009

[50] Worrall DE , Wilson CM , Brostow GJ . Automated retinopathy of prematurity case detection with convolutional neural networks. Carneiro G , Mateus D , Peter L , et al. (eds) Deep Learning and Data Labeling for Medical Applications (DLMIA 2016, LABELS 2016). Cham, Switzerland: Springer; 2016:68‐76. Lecture Notes in Computer Science.

[51] Wentzensen N , Walker JL , Gold MA , et al. Multiple biopsies and detection of cervical cancer precursors at colposcopy. J Clin Oncol. 2015;33(1):83‐89. 25422481 10.1200/JCO.2014.55.9948 PMC4268255

[52] Human Papillomavirus and Related Diseases Report WORLD [Internet]. www.hpvcentre.net. Accessed April 14, 2021.

[53] Schiffman M , Wentzensen N . Issues in optimising and standardising the accuracy and utility of the colposcopic examination in the HPV era. Ecancermedicalscience. 2015;9:530. 25987899 10.3332/ecancer.2015.530 PMC4431398

[54] Dunnmon JA , Yi D , Langlotz CP , Ré C , Rubin DL , Lungren MP . Assessment of convolutional neural networks for automated classification of chest radiographs. Radiology. 2019;290(3):537‐544. 30422093 10.1148/radiol.2018181422 PMC6358056

[55] Chang K , Beers AL , Brink L , et al. Multi‐institutional assessment and crowdsourcing evaluation of deep learning for automated classification of breast density. J Am Coll Radiol. 2020;17(12):1653‐1662. 32592660 10.1016/j.jacr.2020.05.015 PMC10757768

[56] Xue Z , Novetsky AP , Einstein MH , et al. A demonstration of automated visual evaluation of cervical images taken with a smartphone camera. Int J Cancer. 2020;147(9):2416‐2423. 32356305 10.1002/ijc.33029 PMC11977665

[57] Soenksen LR , Kassis T , Conover ST , et al. Using deep learning for dermatologist‐level detection of suspicious pigmented skin lesions from wide‐field images. Sci Transl Med. 2021;13:17. 10.1126/scitranslmed.abb3652 33597262

[58] Ting DSW , Peng L , Varadarajan AV , et al. Deep Learning in ophthalmology: The technical and clinical considerations. Progress in retinal and eye research. Prog Retin Eye Res. 2019;72:100759. 31048019 10.1016/j.preteyeres.2019.04.003

[59] Doshi‐Velez F , Perlis RH . Evaluating machine learning articles. JAMA. 2019;322:1777‐1779. 31714974 10.1001/jama.2019.17304

[60] Zech JR , Badgeley MA , Liu M , Costa AB , Titano JJ , Oermann EK . Variable generalization performance of a deep learning model to detect pneumonia in chest radiographs: a cross‐sectional study. PLoS Med. 2018;15(11):e1002683. 30399157 10.1371/journal.pmed.1002683 PMC6219764

[61] AlBadawy EA , Saha A , Mazurowski MA . Deep learning for segmentation of brain tumors: impact of cross‐institutional training and testing. Med Phys. 2018;45(3):1150‐1158. 29356028 10.1002/mp.12752

[62] Software as a Medical Device (SaMD) Action Plan [Internet]. www.fda.gov. Accessed April 14, 2021.

[63] Deep Learning for the Diagnosis of Stage in Retinopathy of Prematurity: Accuracy and Generalizability across Populations and Cameras. Elsevier Enhanced Reader [Internet]. https://reader.elsevier.com/reader/sd/pii/S2468653020305005?token=6FF5617A436D8199377BA921DC4BF9294E8D32447C2F82531891078E38399E2FE21C3F7D539C26B14DD9967C5F4ED20A&originRegion=us‐east‐1&originCreation=20210414183909. Accessed April 14, 2021. 10.1016/j.oret.2020.12.013 PMC8364291 33561545

[64] Stelzle D , Tanaka LF , Lee KK , et al. Estimates of the global burden of cervical cancer associated with HIV. Lancet Glob Health. 2021;9(2):e161‐e169. 33212031 10.1016/S2214-109X(20)30459-9 PMC7815633

[65] Looker KJ , Rönn MM , Brock PM , et al. Evidence of synergistic relationships between HIV and human papillomavirus (HPV): systematic reviews and meta‐analyses of longitudinal studies of HPV acquisition and clearance by HIV status, and of HIV acquisition by HPV status. J Int AIDS Soc. 2018;21:e25110. 29873885 10.1002/jia2.25110 PMC5989783

[66] Clifford GM , Tully S , Franceschi S . Carcinogenicity of human papillomavirus (HPV) types in HIV‐positive women: a meta‐analysis from HPV infection to cervical cancer. Clin Infect Dis. 2017;64(9):1228‐1235. 28199532 10.1093/cid/cix135 PMC5399941

[67] Ajenifuja KO , Gage JC , Adepiti AC , et al. A population‐based study of visual inspection with acetic acid (VIA) for cervical screening in rural Nigeria. Int J Gynecol Cancer. 2013;23(3):507‐512. 23354369 10.1097/IGC.0b013e318280f395 PMC3580031

[68] Herrero R , Schiffman MH , Bratti C , et al. Design and methods of a population‐based natural history study of cervical neoplasia in a rural province of Costa Rica: the Guanacaste project. Rev Panam Salud Publ. 1997;1(6):411‐425. 10.1590/s1020-49891997000500005 9180057

[69] Shah NH , Milstein A , Bagley SC . Making machine learning models clinically useful. JAMA. 2019;322:1351‐1352. 31393527 10.1001/jama.2019.10306

[70] Beede E , Baylor E , Hersch F , et al. A human‐centered evaluation of a deep learning system deployed in clinics for the detection of diabetic retinopathy. Paper presented at: CHI Conference on Human Factors in Computing System; April 25‐30, 2020; Honolulu, HI.

[71] Google's medical AI was super accurate in a lab. Real life was a different story. MIT Technology Review [Internet]. https://www.technologyreview.com/2020/04/27/1000658/google-medical-ai-accurate-lab-real-life-clinic-covid-diabetes-retina-disease/. Accessed 31 August, 2021.

[72] Porwal P , Pachade S , Kokare M , et al. IDRiD: diabetic retinopathy: segmentation and grading challenge. Med Image Anal. 2020;59:101561. 31671320 10.1016/j.media.2019.101561

[73] Tsiknakis N , Theodoropoulos D , Manikis G , et al. Deep learning for diabetic retinopathy detection and classification based on fundus images: a review. Comput Biol Med. 2021;135:104599. 34247130 10.1016/j.compbiomed.2021.104599

[74] Wentzensen N , Chirenje ZM , Prendiville W . Treatment approaches for women with positive cervical screening results in low‐and middle‐income countries. Prev Med. 2021;144:106439. 33678236 10.1016/j.ypmed.2021.106439

[75] WHO . WHO Guidelines for the Use of Thermal Ablation for Cervical Pre‐Cancer Lesions. http://www.who.int/reproductivehealth/publications/thermal-ablation-for-cervical-pre-cancer-lesions/en/. Accessed April 14, 2021. 31661202

[76] Gage JC , Rodriguez AC , Schiffman M , et al. Treatability by cryotherapy in a screen‐and‐treat strategy. J Low Genit Tract Dis. 2009;13(3):174‐181. 19550216 10.1097/LGT.0b013e3181909f30 PMC2735767

[77] MobileODT . VisualCheck AI [Internet]. MobilleODT. https://www.mobileodt.com/visualcheck/. Accessed November 25, 2020.

[78] Periwinkle Technologies Pvt. Ltd . SMART SCOPE® [Internet]. http://periwinkletech.com/index.php/smart-scope. Accessed November 25, 2020.

