# Supplementary material for: The development of “automated visual evaluation” for cervical cancer screening: The promise and challenges in adapting deep‐learning for clinical testing
Source: Int J Cancer. 2021 Dec 6;150(5):741–52. doi: 10.1002/ijc.33879 (PMC8732320; doi:10.1002/ijc.33879)
Supplement: Supplementary file 1 — Appendix S1 Supporting Information. [file IJC-150-741-s001.pdf]

SUPPLEMENTARY MATERIAL (DIGITAL ONLY)

The development of "Automated Visual Evaluation" for cervical cancer screening: The promise and challenges in adapting deep-learning for clinical testing

Authors

Kanan T. Desai, Brian Befano, Zhiyun Xue, Helen Kelly, Nicole Campos, Didem Egemen, Julia Gage, Ana-Cecilia Rodriguez, Vikrant Sahasrabuddhe, David Levitz, Paul Pearlman, Jose Jeronimo, Sameer Antani, Mark Schiffman, Silvia de Sanjose

Table of Contents

Supplementary Table 1: Checklist to evaluate an AI-based technology (AVE) as part of cervical screening 2

Supplementary Box 1: Clinically relevant epidemiological and technical decisions involved in training AI-based AVE 5

Supplementary Figure 1: Appearance of control versus precancer images: (A) HPV-negative normal, (B) HPV-positive, normal cytology, (C) HPV-positive, ASC-US-cytology, histopathologic low-grade, (D) HPV-positive, HSIL cytology, histopathologic precancer 6

Supplementary Figure 2: Output of a binary AVE classification algorithm on near-duplicate images..... 7

Supplementary Figure 3: Selection of a DL approach 8

Supplementary Figure 4: Reproducibility of AVE 9

Supplementary Figure 5: Guarding against overfitting: creating a more generalizable AVE algorithm. 10

Supplementary Figure 6: Effect of image capture method on cervical image appearance and device portability 11

Supplementary Figure 7: Potential confounding factors, effect modifiers, and distractors of AVE for precancer detection 12

References: 13

Supplementary Table 1: Checklist to evaluate an AI-based technology (AVE) as part of cervical screening

Stage of algorithm development	Area of consideration	Considerations
Before training the algorithm	The indicated use of AVE	✓ Is the proposed AVE algorithm trained for use in general screening, part of triage of HPV-positives, or colposcopy referral population? Accordingly, will the proposed algorithm be useful in one's clinical setting for patients' clinical needs?
	Clarifying target population for using AVE	✓ Is the use of the algorithm clinically meaningful among the intended population? (e.g., What is the age range in which the AVE algorithm claims to identify precancers accurately? Is AVE's performance at extreme ages reported, and is the accurate performance at extreme ages likely due to confounding or bias? Is a negative AVE result in a woman with type III TZ reported with caution in interpreting the score?)
	Aligning the AVE classification categories with the natural history of HPV and cervical carcinogenesis	✓ Does the proposed prediction by the algorithm have a biological rationale & clinical relevance based on the natural history of the disease? (e.g., Is the AVE algorithm trained to discriminate benign HR-HPV infections from histopathological precancer or, is it trained to discriminate arbitrary CIN1-CIN2-CIN3 stages?)<br>✓ Do the predicted class categories lead to risk discrimination for distinct clinical actions? (e.g., no treatment, treatment with ablation only, treatment with ablation or excision)
	Reference standard for defining the AVE classification categories	✓ Is the reference standard used to define the prediction categories a reliable and accurate indicator of the target the algorithm intends to identify? (e.g., Is the histopathology used to define precancer, or is it based only on colposcopic or visual impression?)
Choosing images and metadata for training the algorithm	Size, source, and representativeness of the dataset	✓ How many images in each target category are used to develop an AVE algorithm? Is the size sufficient to train an accurate and generalizable algorithm?<br>✓ Is there a sufficiently big independent hold-back (test) set of images to check the validity?<br>✓ Were ethical considerations given to images used to develop the algorithm? (e.g., Were the images used to develop the algorithm collected under informed consent or approved by an ethical committee? Whenever applicable, were the women aware of their images being used for commercial product development? Will the DL algorithm device be freely available to the women included in developing the algorithm?)

		✓ Does the training include images from a single screening clinic/country or diverse setting?<br>✓ Does the training include images from clinically important subgroups? (e.g., population with a high prevalence of cervicitis, schistosomiasis, HIV)
	Image quality evaluation and pre-exclusion	✓ How does the algorithm perform on low-quality images (i.e., considered as non-evaluable by humans)?<br>✓ Is the prediction by AVE on low-quality images likely to lead to overtreatment or false reassurance?<br>✓ What kind of quality parameters (e.g., blur, shadow, glare etc.) affect AVE performance?<br>✓ Is there a mechanism to evaluate image quality before applying the AVE algorithm? If so, what proportions of images are discarded as non-evaluable due to poor quality?
**Choosing DL methods for training AVE**		✓ Are the DL methods chosen appropriately for the problem at hand? (e.g., multi-class ordinal classification network to identify normal, low-grade, precancer, and cancer)<br>✓ Are the methods reported in a peer-reviewed publication & the images made available open-source for the performance to be verified by an independent group of ML experts?
**Validation of the output of the algorithm**	Reproducibility of AVE	✓ Does the AVE provide the same prediction on the same image tested repeatedly and near-duplicate sets of identical images from a woman?
	Internal validity of AVE	✓ Is the algorithm's performance evaluated on an independent test set not included in either training or validation?<br>✓ Were any cervical images, particularly with equivocal diagnosis (e.g., low-grade), excluded from the test sets? Are the exclusions likely to inflate the overall accuracy of the algorithm?<br>✓ What is the reference standard against which the performance of the algorithm evaluated? (e.g., Is the performance compared to histopathologic truth?)<br>✓ Is the algorithm's performance compared to other existing tests? (e.g., Is the performance superior to VIA as practiced in the particular setting? Is the performance at least non-inferior to expert colposcopic impression?)<br>✓ Is the choice of a given statistic test to assess the performance of AVE appropriate?
	External validity (generalizability) of AVE and avoiding overfitting	✓ Is the algorithm's performance evaluated on more than one test set from diverse settings to examine the generalizability and guard against overfitting?

		✓ Was the evaluation replicated in multiple settings by different groups under formal trial?
	Device portability of AVE	✓ What is the performance of an AVE algorithm on image capture devices not included in the training set?<br>✓ Is the device restriction to use the algorithm explicitly stated in the user manual?
	Anatomical and biologic confounding factors and effect modifiers of AVE	✓ Is AVE's performance evaluated in the clinically relevant subgroups (e.g., WLWH, women infected with cervical schistosomiasis, cervical cervicitis) and explicitly reported? Is it sufficiently different to warrant a subgroup-specific algorithm?
	Risk prediction: "Calibration" of AVE	✓ What is the output of the algorithm, and what does it mean statistically and clinically?<br>✓ Is AVE's outcome calibrated to translate to the probability/risk of a woman having a precancer?
	Predicting immediate versus future risk	✓ What is the duration of reassurance provided by a negative AVE prediction?
**Field Implementation**		✓ How much time is required by the AVE algorithm to provide prediction per image? Is the prediction fast enough to allow the real-time examination of women in the screening clinic?<br>✓ Does the algorithm run on mobile devices without internet connectivity?<br>✓ Is the algorithm specified to be used as a standalone tool or as an assistive technology to human interpretation?<br>✓ Is there a data management system to track women identified as high-risk for precancer by AVE and referred for further evaluation/treatment?<br>✓ Is there a capacity building for treatment of those identifies as high-risk of precancer by AVE?<br>✓ Is there a formal cost-effectiveness analysis to evaluate the impact of AVE?

Supplementary Box 1: Clinically relevant epidemiological and technical decisions involved in training AI-based AVE

- Which type of stain (e.g., acetic acid, Lugol's iodine, or green filter), applied to the cervix provides images with the best contrast for the case-control discrimination by the AVE algorithm and are ideal for AVE development? Does combining multiple stained images per woman improve AVE performance?
- At what time after acetic acid application provides the best contrast in the image for AVE evaluation? Is the uptake rate of acetic acid a piece of additional information contributing significantly to case-control discrimination by AVE? If yes, is a video or image series more appropriate for AVE evaluation than a single still image?
- How to deal with the high imbalance issue between controls and cases in the population (e.g., 99:1 control to case ratio in the general screening population)? Should we use a relatively balanced training set (such as a case to control ratio of 1:1 or 1:2 or 1:3), using significantly smaller numbers of controls than the available controls in the population? Does training on the artificially balanced ratio significantly increase the algorithm's chances for betting on cases over controls? Which algorithms can accommodate a larger ratio of controls to cases in the training and deal with the challenge of data imbalance in the population more effectively?
- Whether including the duplicate images from a single woman's visit on a single device enriches the training set through natural augmentation/enrichment, or should one best image per woman per visit per device be selected? Should both natural and artificial augmentation/enrichment be used?
- Should we combine the patient's textual information (age, HPV status, HIV status, etc.) with images to train the AVE? If so, at which level (e.g., input level, decision level) and how?
- What features is the algorithm looking for to discriminate cases and controls? How to visualize, explain, and interpret the network (i.e., algorithm's prediction)? What is the black box of AI for AVE?
- How well does the model generalize across different devices, different populations, and images captured by different user groups? How to deal with dataset heterogeneity, domain shift, and catastrophic forgetting and how to make the AVE model more robust across these adversaries?

Other AI related complex technical decisions

- Whether to train an AVE using the supervised vs. semi-supervised vs. unsupervised ML method?
- Which general ML framework (or architecture) is more suitable for AVE development work? (e.g., object detection networks such as RetinaNet, CNN-based classification networks such as a ResNet variant, transformer-based classification networks such as ViT, ensemble approaches that combine multiple networks, etc.)
- Whether a transfer learning/ pre-training is helpful for AVE algorithm training? If yes, which database is more helpful (e.g., ImageNet, COCO)? Whether the pre-training based on only medical or cervical images superior to generalized image sets?
- How to choose a good set of hyperparameters (both the type that determines network structure such as number of layers, number of nodes, activation functions in both hidden layers and the output layer, etc. and the type that controls how the network is trained such as learning rate, stopping rules, batch size, regularization parameters, loss functions, etc.) once the specific general ML architecture is decided? To what degree should significant time and efforts be spent to optimize the hyperparameters?
- Whether a separate validation or k-fold cross-validation was used to train and fine-tune the model? How to select the final model or a combination of those models in k-fold cross-validation? How does it affect the AVE performance on a test set?

Supplementary Figure 1: Appearance of control versus precancer images: (A) HPV-negative normal, (B) HPV-positive, normal cytology, (C) HPV-positive, ASC-US-cytology, histopathologic low-grade, (D) HPV-positive, HSIL cytology, histopathologic precancer

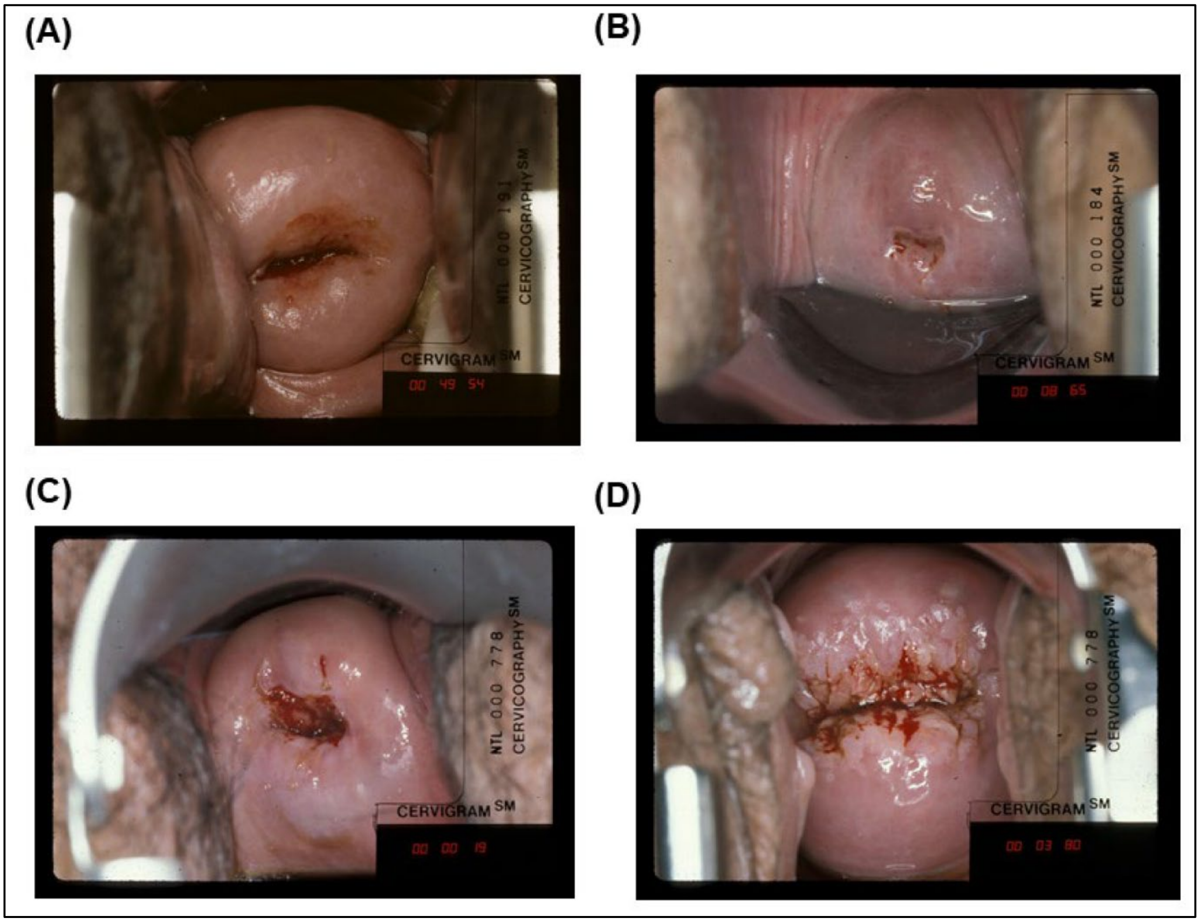

Cervices of women referred to colposcopy (HPV-positive with or without cytologic abnormalities), even when the histopathologic diagnosis is <CIN2, tends to look more severe than cervices of HPV-negative women. The closer the appearance of controls to cases, the larger is the size of the training set needed to develop an algorithm, and the poorer is the case-control distinction achieved by the algorithm. (e.g., an algorithm differentiating HPV-negative control from precancer in a general screening setting achieves better discrimination performance as compared to an algorithm differentiating HPV-positive ASCUS control from precancer in colposcopic triage setting) See also Figure 1 Hence, an algorithm developed for its use in a general screening population, where the percentage of disease people is very low, with more normal-appearing images used for training, if used without further validation in a colposcopy setting is likely to have higher false-positives and vice-versa.
[Cervical image source: Herrero R et al., 1997 (1); ASCUS: Atypical squamous cell of undetermined significance; HSIL: high-grade squamous intraepithelial lesion]

Supplementary Figure 2: Output of a binary AVE classification algorithm on near-duplicate images

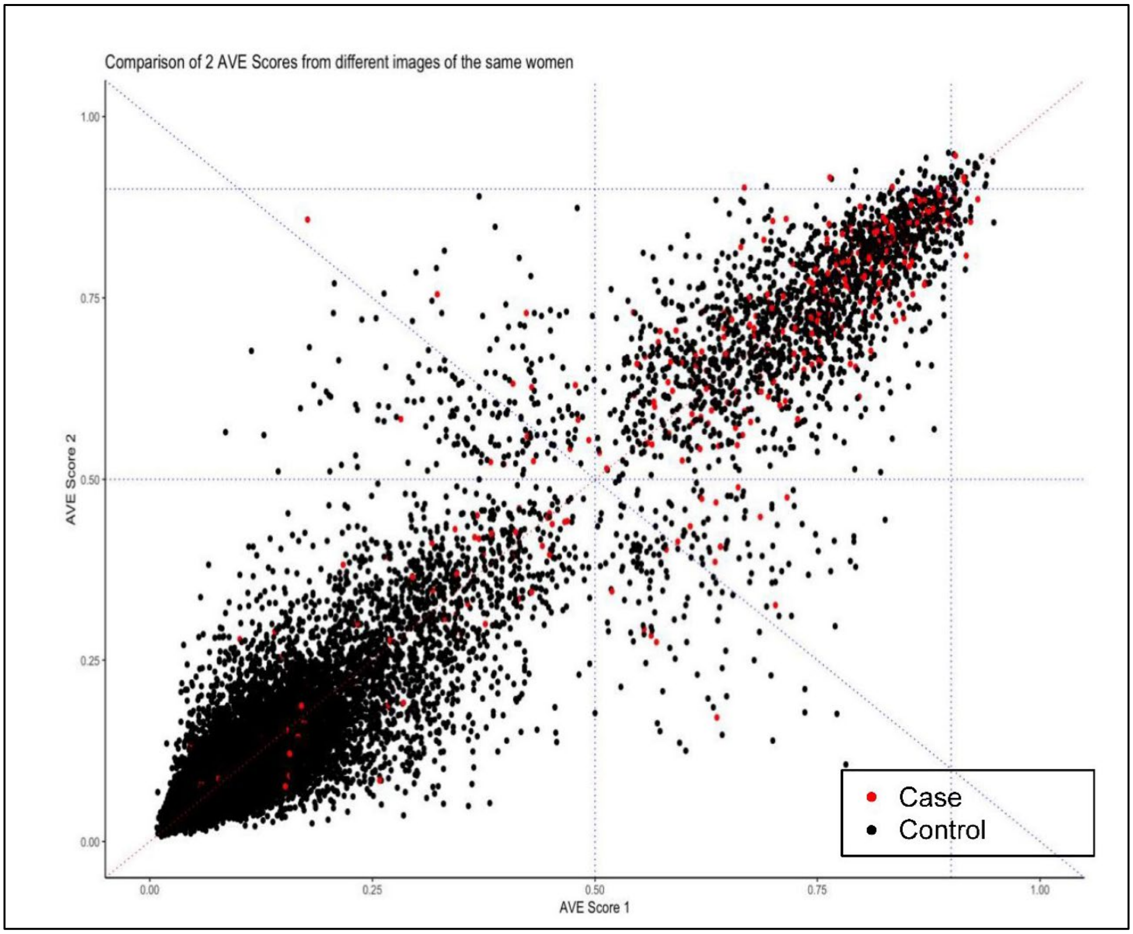

An algorithm trained only on clearest examples of cases and control class, when tested on the images from the intermediate class (i.e., equivocal cases and controls due to low-grade HPV related changes not included in the training), sometimes tends to push the AVE score (for the precancer class) towards extremes (0-certain control or 1-certain case prediction), away from 0.5 (i.e., uncertain prediction). For near-duplicate cervigram images (two images from a single woman collected at the same visit), such an algorithm classifies one of the images as a definite case and the other as definite control. It is important to note that most medical problems need to be classified in ordinal categories (e.g., none, mild, moderate, severe) with an equivocal intermediate zone instead of clear black-white (i.e., case-control) distinction. A DL algorithm, to be applied as a clinical test, should be trained on this multi-class ordinal scale (including equivocal classes) to predict the equivocal changes reliably as a separate category from cases and controls.

[Algorithm source: Binary classification algorithm trained on cervigram images from NHS tested on cervigram images from NHS (unpublished results by NCI HPV-AVE research group)]

Supplementary Figure 3: Selection of a DL approach

Type of DL network	Desired target	Example			
		Question	Input data	Truth label for training	Output on test set
Regression network 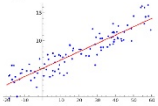	Numeric	What is the probability (risk) of a woman having precancer?	Images: 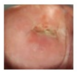 Numerical: HPV type, Age, HPV duration, Cytology results	Probability (prevalent risk) of each woman with the corresponding input data having precancer (P) (e.g., 0.3, 0.5, 1, 0.7)	Probability of each woman in the test set having precancer (e.g., 0.4, 0.6, 0.8, 0.01)
One class classification network 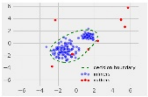	Categorical (one category)	Does the image belong to an outlier class of precancer?	Images (restricted to not precancer ones): 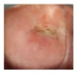	1= Not precancer	0=Precancer (anomaly/outlier class) OR 1= not precancer
Binary class classification network 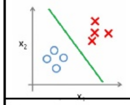	Categorical (two category)	Does the image belong to precancer class or normal class?	Images: 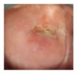	0=Normal class 1=Precancer class	0=Normal class OR 1=Precancer class
Multiclass classification network 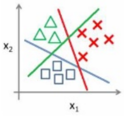	Categorical (more than two category)	Does the image belong to cancer class or precancer class or low-grade class or normal class?	Images: 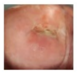	0=Normal class 1=Low-grade class 2=Precancer class 3=Cancer class	0=Normal class OR 1=Low-grade class OR 2=Precancer class OR 3=Cancer class

There is no universal approach to train a DL algorithm. The DL approach chosen in training an algorithm is influenced by the problem one is trying to solve (i.e., desired target class to be predicted) and the availability of truth labels (i.e., training data) to train such a network. One size does not fit all possible questions. The ideal AVE algorithm would provide an absolute risk of precancer for each woman. However, true probability or risk estimates to train such an algorithm are only available from longitudinal studies with long-term follow-up and comprehensive data, not available for widely available image sets. The alternative is perhaps to use a classification network, ideally a multiclass ordinal classification network incorporating low-grade (i.e., HPV-related equivocal changes) as a separate training category.

[Graph source: Terry-Jack M et al., 2019 (5); Gutierrez D et al., 2016 (6); Cervical image source: Desai K et al., 2020 (3)]

Supplementary Figure 4: Reproducibility of AVE

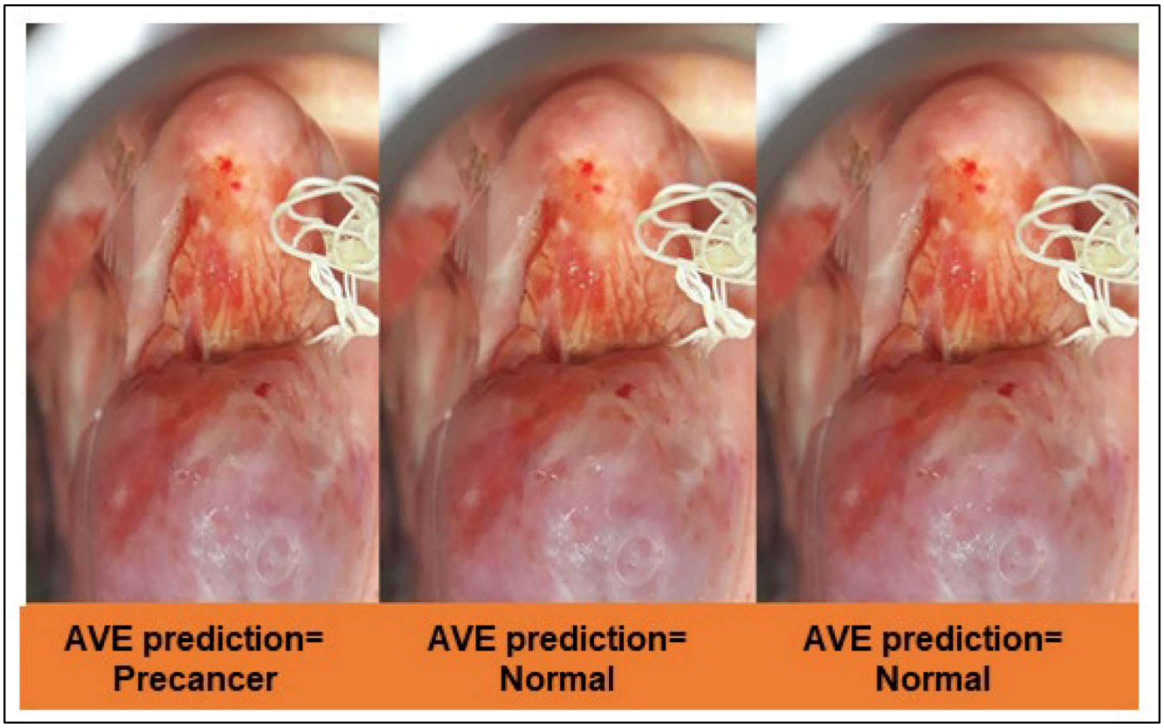

A given woman on a particular visit 'has' or 'has not' a precancer and her management on a given day will be based on a test to detect her status reliably and accurately. Visually identical appearing images collected from a single woman on a single visit with a single device, may have subtle changes at the image pixel levels due to changes in woman's body position, inspiration-expiration status, etc. This may lead an algorithm to predict different outputs for such near-duplicate images from a single woman. However, such a fragile or non-robust algorithm is imprecise, and so it can never be accurate and accepted as a clinical test.
[Algorithm source: Binary classification algorithm trained on EVA images globally (Zhiyun X et al., 2020 (2)), tested on EVA images from Project Itoju, Nigeria (unpublished results by NCI HPV-AVE research group); Cervical image source: Desai K et al., 2020 (3)]

Supplementary Figure 5: Guarding against overfitting: creating a more generalizable AVE algorithm

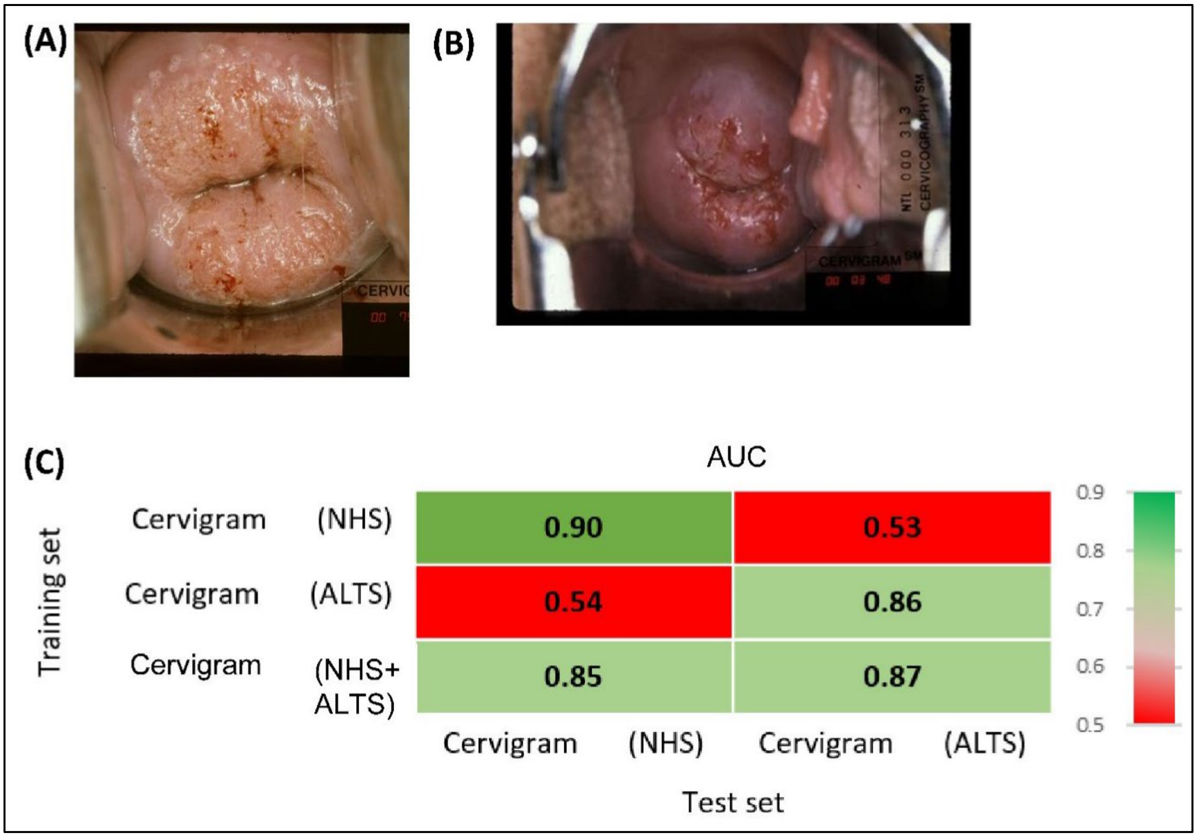

The image in a) is a cervigram from the Guanacaste Natural history Study (NHS) and b) is a cervigram from the ASC-US LSIL triage study (ALTS). c) An AVE algorithm trained on cervigram images from NHS may be overfitted to the image features and patient characteristics from that setting, yielding in this example an AUC= 0.90. When this algorithm is applied to test set of images from ALTS, the AUC drops to 0.53. To improve the performance some images from the ALTS study were added to the NIH training set. As a result, the combinedly trained algorithm from NHS and ALTS images provided an AUC of 0.85 on NHS test images and very similar AUC=0.87 on ALTS test images. Therefore, it is always important to verify the algorithm's performance on test set of images from diverse clinics in addition to the test set of images from the same database used for training.

[Algorithm source: c1) Binary classification algorithm trained on cervigram images from NHS tested on cervigram images from NHS, c2) Binary classification algorithm trained on cervigram images from ALTS tested on cervigram images from ALTS, c3) Binary classification algorithm trained on cervigram images from NHS+ALTS tested on cervigram images from NHS+ALTS (unpublished results by NCI HPV-AVE research group); Cervical image source: Herrero R et al., 1997 (1) (a), Schiffman M et al., 2000 (7) (b)]

Supplementary Figure 6: Effect of image capture method on cervical image appearance and device portability

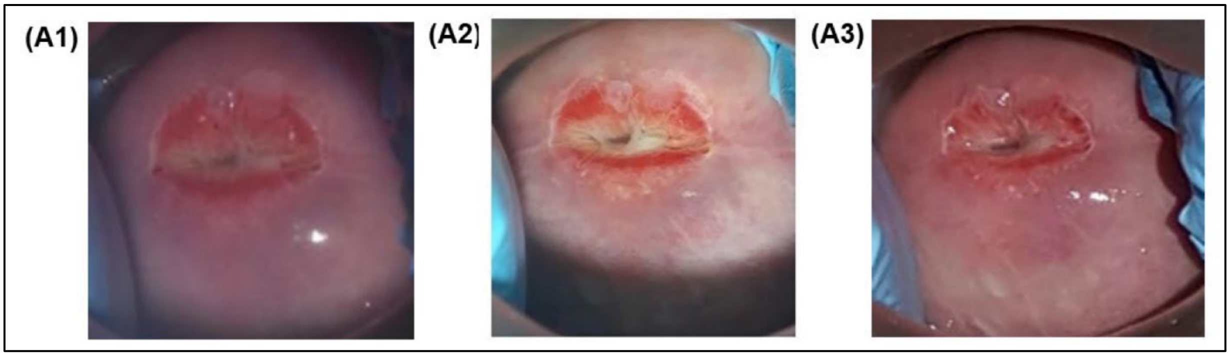

The appearance of the cervix when captured in the same woman with different image capture devices [e.g., (A1) Zeiss colposcope with a DSLR camera and beam splitter, (A2) EVA system (enhanced J5 cellphone with extra light and lens, (A3) Samsung S8 smartphone] has variation in color, glare, brightness, and other features, which makes portability of an algorithm trained on one kind of camera image to a different kind of camera image a challenge.
[Cervical image source: Desai K et al., 2020 (3); DSLR-Digital Single Lens Reflex]

Supplementary Figure 7: Potential confounding factors, effect modifiers, and distractors of AVE for precancer detection

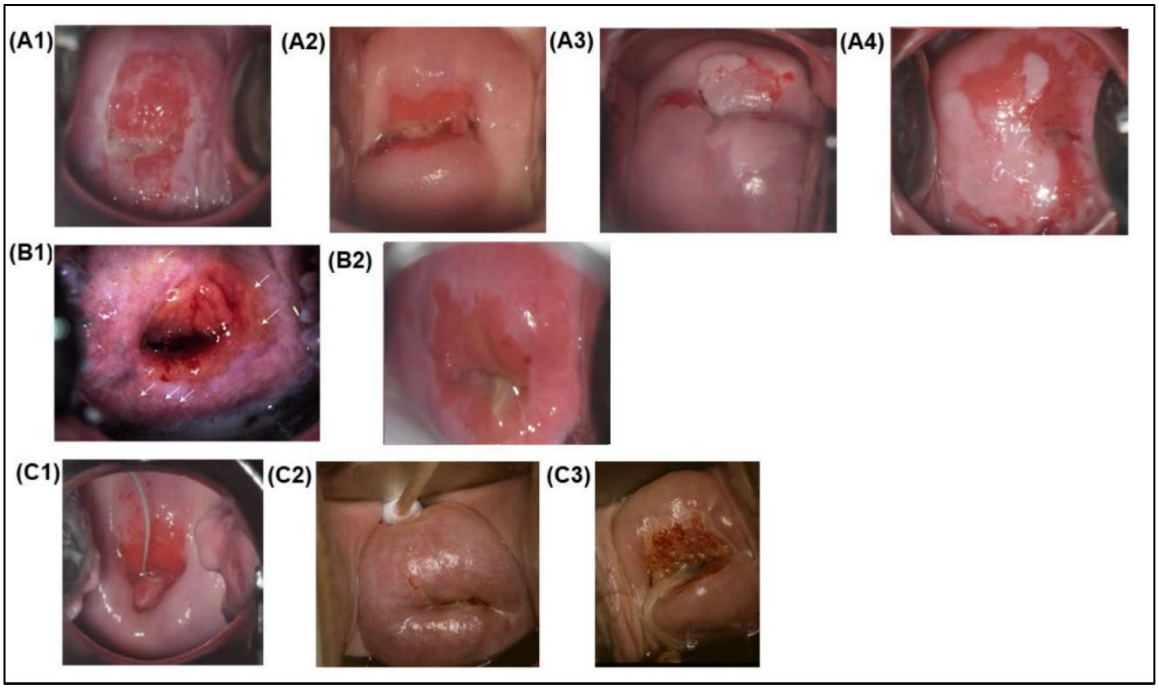

(A) Histopathologic precancer and controls among WLWH tend to appear more severe than among HIV-negative women [e.g., (A1) HIV-negative precancer, (A2) HIV-negative, HPV-positive low-grade (control), (A3) HIV-positive precancer, (A4) HIV-positive HPV-positive low-grade (control)] making the case-control discrimination by AVE harder in WLWH. The performance of AVE must be verified in the WLWH subgroup, and if found to be significantly different than in HIV-negative women, then a sub-group specific algorithm may need to be developed for WLHIV

(B) Certain other conditions [e.g., (B1) Cervical schistosomiasis, (B2) Cervicitis] may lead to false-positive AVE due to their appearance on the cervix. These conditions are not associated directly with cervical precancer and hence could be potential confounding factors of AVE

(C) Additionally, there are some artifacts that are routinely encountered and are unavoidable in the clinical practice (e.g., (C1) Intrauterine device string, (C2) Scopate, (C3) Thick mucus plug). If these are present outside the ROI (i.e., SCJ), their effect could be partly mitigated by creating a smaller bounding box (i.e., cropping). However, if these are present within the SCJ, then the AVE algorithm needs to be trained & tested to be robust enough not to be distracted by these factors.

[Cervical image source: Desai K et al., 2020 (3); Herrero R et al., 1997 (1); Norseth HM et al., 2014 (8)]

References:

1. Herrero R, Schiffman MH, Bratti C, Hildesheim A, Balmaceda I, Sherman ME, et al. Design and methods of a population-based natural history study of cervical neoplasia in a rural province of Costa Rica: The Guanacaste Project. Rev Panam Salud Publica/Pan Am J Public Heal [Internet]. 1997 [cited 2021 Feb 4];1(6):411–25. Available from: https://pubmed.ncbi.nlm.nih.gov/9180057/
2. Xue Z, Novetsky AP, Einstein MH, Marcus JZ, Befano B, Guo P, et al. A demonstration of automated visual evaluation of cervical images taken with a smartphone camera. Int J Cancer [Internet]. 2020 Nov 1 [cited 2021 Jun 21];147(9):2416–23. Available from: https://www.mobileodt.com/eva-system
3. Desai KT, Ajenifuja KO, Banjo A, Adepiti AC, Novetsky AP, Sebag C, et al. Design and feasibility of a novel program of cervical screening in Nigeria: self-sampled HPV testing paired with visual triage. Infect Agents Cancer. 2020;
4. Long LR. Introduction to Neural Networks and Deep Learning (unpublished). Bethesda, MD; 2021.
5. Tips and Tricks for Multi-Class Classification | by Mohammed Terry-Jack | Medium [Internet]. [cited 2021 Mar 13]. Available from: https://medium.com/@b.terryjack/tips-and-tricks-for-multi-class-classification-c184ae1c8ffc
6. How to Use Isolation Forests for Anomaly Detection - insideBIGDATA [Internet]. [cited 2021 Mar 13]. Available from: https://insidebigdata.com/2016/11/11/how-to-use-isolation-forests-for-anomaly-detection/
7. Schiffman M, Adrianza ME. ASCUS-LSIL Triage Study: Design, methods and characteristics of trial participants. Acta Cytol [Internet]. 2000 [cited 2021 Jun 22];44(5):726–42. Available from: https://pubmed.ncbi.nlm.nih.gov/11015972/
8. Norseth HM, Ndhlovu PD, Kleppa E, Randrianasolo BS, Jourdan PM, Roald B, et al. The Colposcopic Atlas of Schistosomiasis in the Lower Female Genital Tract Based on Studies in Malawi, Zimbabwe, Madagascar and South Africa. [cited 2021 Mar 13]; Available from: www.plosntds.org
